# Salt Stress Mitigation and Field-Relevant Biostimulant Activity of Prosystemin Protein Fragments: Novel Tools for Cutting-Edge Solutions in Agriculture

**DOI:** 10.3390/plants14152411

**Published:** 2025-08-04

**Authors:** Martina Chiara Criscuolo, Raffaele Magliulo, Valeria Castaldi, Valerio Cirillo, Claudio Cristiani, Andrea Negroni, Anna Maria Aprile, Donata Molisso, Martina Buonanno, Davide Esposito, Emma Langella, Simona Maria Monti, Rosa Rao

**Affiliations:** 1 Department of Agricultural Sciences, University of Naples Federico II, 80055 Portici, Italy; martinachiara.criscuolo@unina.it (M.C.C.); raffaele.magliulo@unina.it (R.M.); valeria.castaldi@yale.edu (V.C.); valerio.cirillo@unina.it (V.C.); anna.aprile@materias.it (A.M.A.); donata.molisso1989m@gmail.com (D.M.); 2 Consorzi Agrari D’Italia, San Giorgio di Piano via Centese 5/3, 40016 Bologna, Italy; claudio.cristiani@consorziagrariditalia.it (C.C.); andrea.negroni@consorziagrariditalia.it (A.N.); 3 Materias Srl, Corso Nicolangelo Protopisani 50, 80146 Naples, Italy; 4 Institute of Biostructures and Bioimaging, National Research Council (IBB-CNR), via Pietro Castellino 111, 80131 Naples, Italy; martina.buonanno@cnr.it (M.B.); davide.esposito@cnr.it (D.E.); emma.langella@cnr.it (E.L.); simonamaria.monti@cnr.it (S.M.M.)

**Keywords:** protein fragments, bioactive peptides, salt stress, field trials, tomato, sustainability, protein repeats, yield, quality

## Abstract

In an increasingly challenging agricultural environment, the identification of novel tools for protecting crops from stress agents while securing marketable production is a key objective. Here we investigated the effects of three previously characterized Prosystemin-derived functional peptide fragments as protective agents against salt stress and as biostimulants modulating tomato yield and quality traits. The treatments of tomato plants with femtomolar amounts of the peptides alleviated salt stress symptoms, likely due to an increase in root biomass up to 18% and the upregulation of key antioxidant genes such as APX2 and HSP90. In addition, the peptides exhibited biostimulant activity, significantly improving root area (up to 10%) and shoot growth (up to 9%). We validated such activities through two-year field trials carried out on industrial tomato crops. Peptide treatments confirmed their biostimulant effects, leading to a nearly 50% increase in marketable production compared to a commonly used commercial product and consistently enhancing fruit °Brix values.

## 1. Introduction

Plants, as sessile organisms, must directly face multiple and constant stresses. To counteract these continuous challenges, they have developed sophisticated chemical-based defense and signaling mechanisms [1]. The output of these mechanisms allows plants to react against adverse growth conditions by triggering the production of primary and secondary danger signals, which help to ward off pest invasions and reduce the damage of other environmental stress agents. Secondary signals, also referred to as phytocytokines, are processed in response to damage [2]. Systemin (Sys) is one of the first examples identified in tomato plants [3,4]. Embedded in the C-terminus of its precursor Prosystemin (ProSys), Sys is released following wounding or herbivore feeding [5]. After its release, it is detected by its specific receptor SYR1, a leucine-rich repeat receptor kinase (LRR-RK) [6]. This interaction, in turn, triggers the production of its precursor, along with the synthesis of jasmonic acid (JA) and the emission of volatile organic compounds (VOCs) [7].

The investigation of Sys activity highlighted a link between biotic and abiotic responses: tomato plants treated with Sys or overproducing Sys showed a cross-tolerant phenotype [8,9]. We recently discovered that Sys is not the only bioactive sequence enclosed in ProSys as two protein fragments located in its N-terminus, named PS1-70 and PS1-120, also exhibit biological activity inducing the expression of defense-related genes and providing protection against *S. littoralis* larvae, as well as *B. cinerea* and *A. alternata* infections, without exerting any direct toxic effects on either target or non-target organisms [10]. Furthermore, recent analysis on the whole ProSys sequence revealed the presence of short repeat motifs (RMs), indicated as G1-4, R1-4 and T1-4, that also confer protection to tomato plants against biotic stressors when exogenously applied [11].

Natural plant peptides are a class of key signaling molecules that have gained great interest as sustainable tools for environmentally responsible crop management. They are induced in response to various stresses, including drought and salinity [12], and function as plant growth regulators, highlighting their versatile potential [13]. Several of these peptides are released by large precursor proteins consisting of 100 amino acids or more through proteolytic processes. These processes occur soon after the challenge to make rapidly available signals that prepare plants to counteract the incoming stress. In addition, plant peptides have also turned out to be a class of hormone molecules [14], able to act locally and systemically at extremely low concentrations (femtomolar to picomolar) to regulate plant stress responses and development [15,16].

Here we investigated the biological activity of different ProSys-derived sequences against salt stress in tomato plants. Our results demonstrated that plants exogenously treated with PS1-70 and PS1-120 fragments, as well as a G1 repeat, have a better performance both with and without salt stress conditions compared to untreated controls, likely as a direct consequence of gene activation following their biostimulant activity. This ability was also validated in field trials, during which treated plants showed an increased total and marketable production.

## 2. Results

### 2.1. Experimental Fragments Improve Root Growth Under Salinity and Act as Shoot Biostimulant

**Figure 1:**
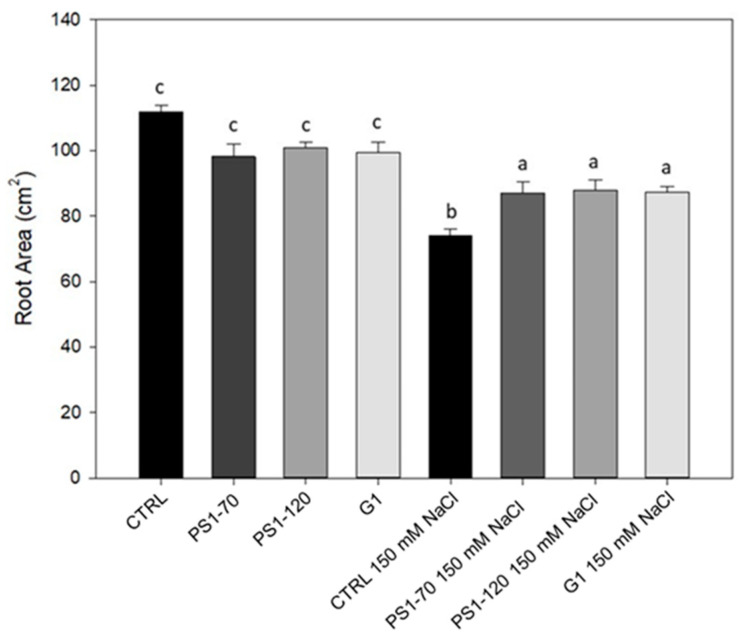
Root area of tomato plants treated with PS1-70, PS1-120 and G1. Tomato root area of plants treated with 100 fM EFs in absence and in presence of salt stress. Error bars indicate standard error. Different letters indicate significant differences according to Duncan post hoc test (*p* < 0.05).

In order to evaluate the potential of exogenous applications of protein fragments PS1-70, PS1-120 and G1 repeat, hereafter referred to as experimental fragments (EFs), to improve tomato growth under salt stress conditions, shoot fresh weight (SFW) and root area were analyzed. Plants were drenched once with 100 fM solutions of each EF and subsequently exposed to salt stress. Our results (Table 1) confirm that salt stress treatment strongly suppresses both SFW and root area. Specifically, the application of 150 mM NaCl caused a 24% decrease in SFW and a 19% decrease in root area compared to zero salt treatment. Meanwhile, EF applications in non-stressed plants significantly enhanced growth: PS1-70 and G1 treatments increased SFW by 9% and 8%, respectively, over the untreated control, while PS1-120 showed no significant change. When EFs were applied to plants exposed to salt stress, SFW did not exhibit a significant salinity–EF interaction; meanwhile, root area displayed a highly significant interaction, as also depicted in Figure 1.

In Figure 1, it is possible to observe that the treatment with EFs did not affect root growth in the absence of salt stress, showing unchanged root area compared with the controls. Under salt stress conditions, the roots of EF-treated plants showed a significant increase in their biomass (+17%, +18% and +18% respectively after PS1-70, PS1-120 and G1 treatment), in comparison with the control, suggesting that the treatment mitigated salt stress.

### 2.2. EFs Positively Affect Stomatal Density and Area

The effect of EF applications was assessed by observing the stomatal density (SD) and area (SA), which may decrease in the presence of salt stress [17]. The analysis of SD and SA studies was conducted using image analysis on leaf impressions. This method emphasizes the micro-morphology of leaf cells by capturing images through light microscopy and subsequent computational processing.

**Figure 2:**
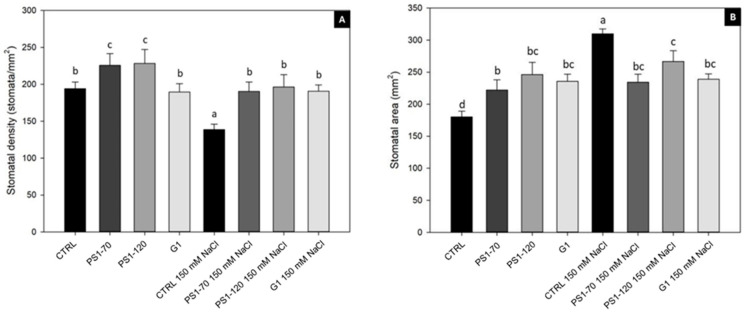
Stomatal density and area of tomato leaves treated with EFs. Stomatal density (**A**) and area (**B**) of tomato leaves treated with 100 fM PS1-70, PS1-120 and G1 in the absence and presence of salt stress. Error bars indicate standard error. A two-way ANOVA, Tukey’s test (*p* < 0.05) was conducted. Letters indicate statistically significant differences between the experimental groups.

In the absence of salt, PS1-70 and PS1-120 induced a significant increase in SD (+16% and +17% respectively), as shown in Figure 2A and in Supplementary Material Figure S1. Notably, irrigations with NaCl caused an SD decrease (−28%) in control plants, while treated plants kept a higher level of SD both in the presence and absence of salt (Figure 2A).

In the absence of salt, SA increased in fragment-treated plants in comparison with untreated controls (+23%, +36% and +30% for PS1-70, PS1-120 and G1 treatment, respectively). Conversely, under salt stress conditions, a significant increase in SA was registered in control plants (+72%) (Figure 2B). In EF-treated samples, a reduction in SA was observed (−24%, −13% and −22% after PS1-70, PS1-120 and G1 treatment, respectively); values closer to the SA value of the non-salinized control were reached, suggesting that ProSys-derived fragments help plants to recover the standard pattern of SA (Figure 2B).

### 2.3. Proline Content Is Reduced by EF Treatments During Salinity Stress

**Figure 3:**
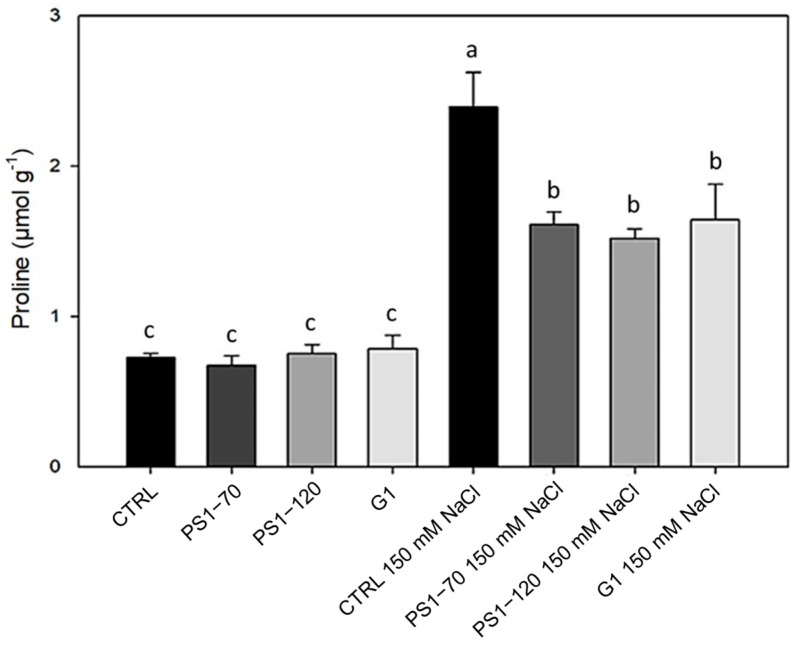
Proline content in plants treated with EFs. Proline content in plants treated with 100 fM PS1-70, PS1-120 and G1 in the absence and presence of salt stress. Error bars indicate standard error. A two-way ANOVA and Tukey’s test (*p* < 0.05) were conducted. Letters indicate statistically significant differences between the experimental groups.

Proline contributes to the adaptation to salt stress as an activator of downstream signal transduction pathways and as an intermediary signaling effector that controls a variety of physiological and metabolic responses, including detoxification of reactive oxygen species (ROS) [18]. EF-treated plants did not influence proline content in the absence of salt, as shown in Figure 3. As expected, in salt-stressed plants, a significant increase in proline content was evident in all samples. Specifically, the presence of 150 mM NaCl caused a marked increase in proline in control plants (+228%). This content was lowered upon EF treatments to +121%, + 109% and +157% when stressed plants were treated with PS1-70, PS1-120 and G1, respectively (Figure 3).

### 2.4. EFs Modulate the Antioxidant Response of the Plant

**Figure 4:**
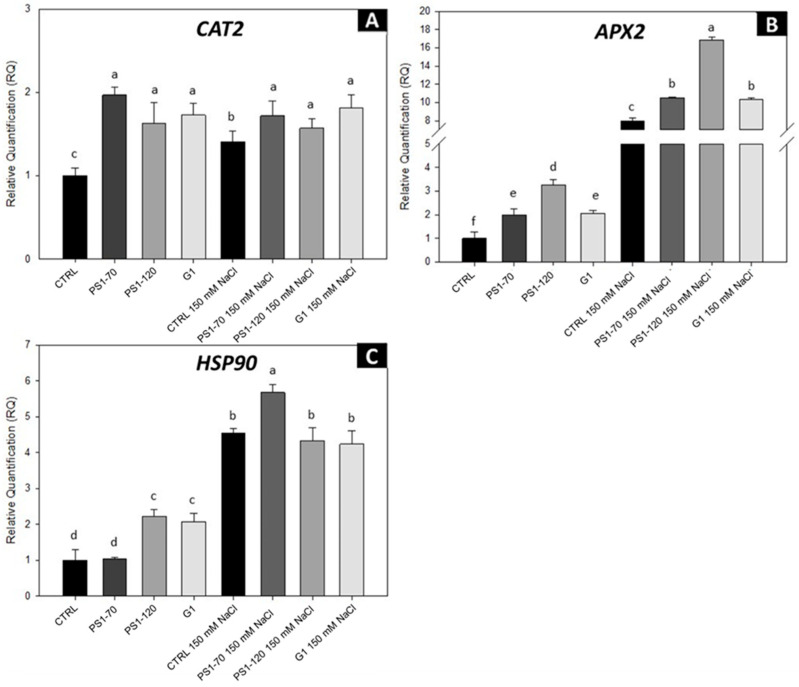
Gene expression analysis of defense-related genes of tomato plants treated with EFs. Relative expression of CAT2 (**A**), APX2 (**B**) and HSP90 (**C**) by RT-PCR treated with 100 fM PS1-70, PS1-120 and G1 in the absence and presence of salt stress. Error bars indicate standard error. Quantities are relative to the calibrator represented by mock-treated plants. Different letters indicate significant differences according to Tukey’s post hoc test (*p* < 0.05). CTRL, control plants.

The antioxidative response, where ascorbate peroxidase (APX) and catalase (CAT2) are key enzymes, is an integral part of the plant tolerance response to environmental stresses. Other proteins with a key role in stress conditions are heat shock proteins, which are very conserved and abundant molecular chaperones [19]. Thus, we analyzed the expression of two antioxidant enzymes, catalase (CAT2, Solyc02g082760.3) and ascorbate peroxidase (APX2, Solyc06g005150), along with a heat shock protein (HSP90, Solyc06g036290), through RT-PCR. As expected, plants treated with EFs in the absence of salt stress triggered a clear upregulation of all three investigated genes, suggesting that their exogenous application is perceived as stress signals priming the plant defense system (Figure 4). Consistent with this observation, transcript levels remained constant for the CAT2 gene (Figure 4A). Interestingly, APX2 and HSP90 genes increased further in the presence of salt stress, suggesting a synergistic interaction in which EFs not only prime defense gene induction but also amplify plants’ stress response under salt stress (Figure 4B,C).

### 2.5. EFs Exhibit Biostimulant Effects on Tomato Plants in Open-Field Experiments

**Figure 5:**
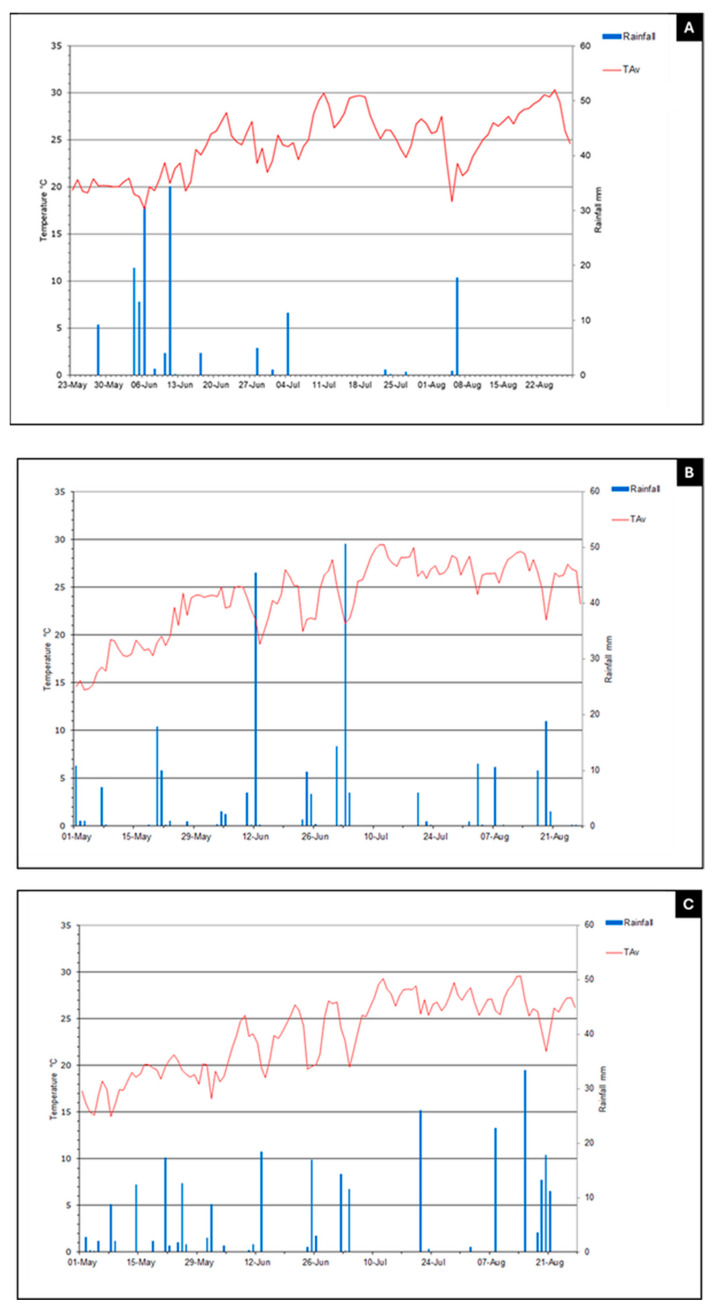
Daily temperatures and rain events during 2023 ((**A**) Experiment 1) and 2024 ((**B**) Experiment 2a and 2b; (**C**) Experiment 3) open-field trials. Blue lines indicate rainfall (mm); red lines indicate Tav (annual average ambient temperature, °C).

Based on the data collected in the current study, along with previously reported findings regarding ProSys fragments [10] and RMs [11], we selected the most promising peptides for the evaluation of their potential biostimulant effects to be used in open-field trials. To this end, we tested PS1-70 and G1 over two years: 2023 (Experiment 1) and 2024 (Experiments 2a, 2b and 3). During the 2023 trial, the maximum and minimum daily temperatures averaged over the cultivation period were 30 °C and 18 °C in 2023 (23 May–22 August), with rainfall occurring predominantly in May and June, with a maximum recorded precipitation of more than 30 mm (Figure 5). These conditions supported the proper development of tomato plants. In 2024, three trials were performed on two different farms, and they were characterized by the maximum and minimum daily temperatures of 29.5 °C and 14.8 °C in both locations and a maximum recorded precipitation of 50 and 34 mm for exp2 and 3, respectively (1 May–21 August) (Figure 5).

**Figure 6:**
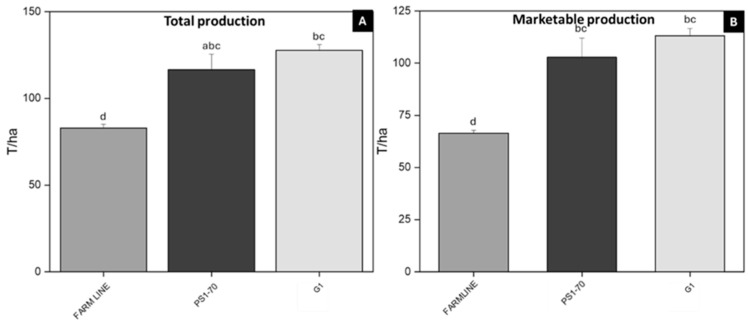
Total and marketable production of tomato plants treated with PS1-70 and G1. Total (**A**) and marketable (**B**) production of plants treated with 100 fM solutions of PS1-70 and G1 in open-field trials (Lagosanto, Ferrara 2024, experiment 2a). Error bars indicate standard error. Means were compared using the Student–Newman–Keuls (SNK) test (*p* ≤ 0.05). Letters indicate different statistical groups.

These conditions were favorable for crop development as well. In the subsequent months, rising temperatures and limited rainfall did not create optimal conditions for disease development. To assess crop performance and, consequently, economic viability parameters that are directly influenced by the application of biostimulants, total and commercial crop production were measured. The open-field trial, carried out in Lagosanto (Ferrara) in 2024 (Exp.2a), was conducted by treating tomato plants every 30 days for a total of three applications. Treatments demonstrated a significant increase in total production for both peptides. Specifically, PS1-70 and G1 treatments resulted in total production increases of 40.64% and 54.13%, respectively (Figure 6A), compared to farm line plants, which are internal control plants treated with commercial fertilizer and defense products. Interestingly, in terms of marketable production, PS1-70 and G1 treatments resulted in remarkable improvements compared to the farm line plants, with increases of 55% and 70%, respectively (Figure 6B).

Across all trials, PS1-70 and G1 demonstrated interesting potential to enhance plant productivity (Supplementary Material Figures S2–S4). In particular, PS1-70 registered increases of 50.4% (total) and 70% (marketable) in Exp.2b (Supplementary Material Figure S3) and increases of 30.4% (total) and 45.7% (marketable) in Exp.3 (Supplementary Material Figure S4). Regarding plants treated with G1 peptide, significant improvements with increases of 69.7% and 54.6% for total and marketable production in exp2b (Supplementary Material Figure S3) and increases of 16.37% (total) and 27.34% (marketable) in exp3 were registered (Supplementary Material Figure S4).

In 2023 trials (Supplementary Material Figure S2), plants treated with PS1-70 and G1 registered significant differences from the untreated plants for both total (+35% and +15% respectively) and marketable production (+46.4% and +14.3%, respectively). Additionally, plants treated with PS1-70 exhibited a slight increase compared to farm line plants of +3.8% and +2.5% for total and marketable production, respectively. No increases were registered for G1 when compared to farm line plants.

Production increases are particularly significant when they directly correlate with higher gross marketable production (GMP) values and higher °Brix levels, indicating that treatments not only enhance yield but also improve fruit quality, underscoring the economic relevance of these treatments. In Table 2, the direct correlation between the increase in product yield and the corresponding GMP values of the field trials conducted in Lagosanto in 2024 (Exp.2a) is reported. Results demonstrate a proportional enhancement in GMP of 27.4% and 45.8% for G1 and PS1-70, respectively, indicating that the application of these peptides not only improves yield parameters (total and marketable production) but also ensures greater economic benefits for commercial farming operations. Additional trials further confirmed this trend, with observed GMP increases of 41% and 69.67% for G1 and P1-70 treated plants in exp.2b and increases of 27.4% and 45.8% for plants treated with G1 and PS1-70, respectively, in exp.3. In 2023 (exp.1) P1-70 registered a higher GMP value of 4.8% (Supplementary Material Table S1).

The °Brix value is a crucial quality parameter in tomato production, as it measures the total soluble solids content (primarily sugars) and provides an indication of fruit ripeness and flavor intensity [20]. Across all experiments, treatments with PS1-70 and G1 consistently enhanced fruit °Brix values compared to internal controls. Detailed results from individual trials are provided in Supplementary Material Table S2. These consistent improvements in sugar content across multiple trials demonstrate the reliable positive effect of peptide treatments on fruit quality parameters, suggesting potential benefits for fruit organoleptic properties and market value.

Furthermore, Figure 5 shows that in 2024, rainfall was distributed uniformly during the whole trial (from transplanting to harvesting) in two locations (Budrio and Lagosanto), ensuring a constant water supply for the entire production cycle of the crop. Conversely, in 2023 in San Giovanni in Persiceto, rainfall was concentrated in the first month between the end of May and mid-June, dropping drastically in the following months (July and August). In addition, during 2023, weather conditions registered high night-time temperatures, while in 2024, temperatures were slightly lower. These trends, despite the crop having an irrigation system, could have affected the crop’s development. It is worth noting that we did not observe large variability among replications of the same thesis in both years and among the Experiments (1, 2a, 2b and 3); thus, the statistical analysis appeared not to be influenced by large variations.

## 3. Discussion

Climate change, resulting from natural phenomena and human activities [21], can significantly impact soil salinity [22], leading to a reduction in crop yield. Consequently, identification of solutions that mitigate these effects is a priority in modern agriculture [23]. It has been demonstrated that peptides or protein fragments enclosed in larger precursors play an important role in the coordination of plant responses to various biotic and abiotic stresses [12]. These fragments, including peptides and amino acids, can also confer positive effects on the physiological processes of different plant species under abiotic and biotic stresses [24]. Arabidopsis Pep1 and Pep3, found in A. Thaliana, are well-characterized plant peptides included in large precursors. In particular, Arabidopsis Pep1 is composed of 23 amino acids and originates from the cleavage of a 92-amino-acid precursor protein. This peptide enhances plant resistance against root (filamentous fungi) and leaf parasites (hemibiotrophic bacteria and necrotrophic fungi) [25,26,27]. Arabidopsis Pep3 is a 30-amino-acid peptide that derives from a 96-amino-acid precursor; it triggers components of the innate immune system and improves plants’ tolerance to high salinity [28]. Specifically, Pep1 triggers the immune signals [29], while Pep3 is highly induced by salt stress, within minutes to hours [28]. ProSys represents a compelling model currently under investigation in our laboratories. This prohormone has been shown not only to release the hormonal peptide Sys [5], but also, as recently demonstrated, to contain additional biologically active sequences that are repeated within its primary sequence [11]. Notably, some of these sequences have been detected in vivo [11]. ProSys appears to interact with several different proteins underpinning multiple plant stress-related activities [30]. Indeed, its interaction plasticity, favored by its disordered features [31], enables ProSys to cope with plant responses to both biotic and abiotic stresses. Here, we expanded the spectrum of functions performed by three ProSys-derived protein fragments previously identified as biologically active (PS1-70, PS1-120, G1) to their ability to promote resilience against salt stress in tomato plants.

We observed that other than the biostimulant effect on plant shoot fresh weight (Table 1), a single irrigation with EFs markedly reduced salt damage, making plants more tolerant. Indeed, treated plants have shown a significant improvement in root area under salt stress conditions. This result is in line with what Orsini et al. observed in plants overexpressing ProSys, where the authors showed that, in response to salt stress, transgenic plants maintained a higher stomatal conductance compared to the wild type. Furthermore, leaf concentrations of abscisic acid and proline were lower in stressed transgenic plants than in wild-type plants [9]. These results suggest that the former either perceived a less stressful environment or adapted more efficiently to it. In addition, under salt stress, ProSys transgenic plants produced higher biomass. Here, our results suggest that salt tolerance cannot be attributed exclusively to an overproduction of Sys but must be attributed to the ability of other regions of the precursor, such as those tested in the present work. These regions appear to be able to reduce the damages of salt stress, likely by integrating multiple signals. Indeed, it has been demonstrated that stress-related pathways are not necessarily independent, but rather characterized by an intricate crosstalk [32,33]. This complex crosstalk involves various phytohormones that collectively regulate genes crucial for hormone biosynthesis and signaling pathways, allowing plants to develop enhanced tolerance to multiple environmental stresses through the overlapping of defense response pathways [34]. Salt and wounding stress, for example, appear to be interconnected by the oxidative burst involving calcium ions and by the production of JA and Sys [9,35]. The key elements that may mediate this crosstalk are calmodulins (CMs) and calcium-dependent protein kinases (CDPKs) [32,36], which interestingly appear to take part in the ProSys interaction network [33].

Abiotic stress, like salt stress, reduces water uptake, disrupting plant physiological processes and producing ionic and osmotic stresses as well as oxidative damage as a consequence of a plethora of stress-induced ROS. High levels of ROS alter cell structure and degrade proteins and nucleic acids [37]. Thus, reducing stress-induced ROS over-accumulation is important to protect plants under adverse environments. Our results show that EFs increase transcripts associated with antioxidative activity both in the absence and in the presence of salt, suggesting a role in shaping plant oxidative status with a reduction in ROS accumulation. These results correlate with the improved performance of salinized plants, showing an increased value in their shoot and in their stomatal density. It was demonstrated that a salt-tolerant pepper genotype showed a much higher level of transcripts of CAT2, APX2 and other antioxidant genes compared to a susceptible genotype when subjected to salt stress [38], suggesting a correlation between ROS scavengers and plant performance in salt stress conditions. Moreover, increased root biomass is often associated with an enhanced tolerance to salinity and drought stresses, and in this context, an important role is played by HSP90 [39], which is also upregulated following plant treatments with specific EFs. For example, tobacco and tomato plants overexpressing HSP90 were more tolerant to salt stress, and their roots grew faster than the wild types [39,40]. It was proposed that HSP90 plays a vital role in improving the salinity tolerance by enhancing the root biomass and architecture [39]. Although salt stress causes a decrease in stomatal density as a strategy to reduce water loss [41,42], plants treated with PS1-70, PS1-120 and G1 do not show such a reduction (Figure 2). Reduced stomatal density, influenced by various interconnected morpho-physiological and metabolic factors, enhances salinity tolerance and water-use efficiency during salt stress. Nevertheless, it was reported that in tomato, this reduction also leads to decreased photosynthetic efficiency, ultimately resulting in lower plant biomass production [43,44,45]. However, the application of the EFs, both in control and in salinized plants, registered higher stomata density than in controls. Such an increase likely allows tomato plants to restore gas exchange, reducing ROS accumulation [46,47]. It is worth noting that an increased stomatal density is associated with increased salinity tolerance in barley [48].

Salt stress induces not only antioxidants but also the accumulation of proline [49]. Proline plays several roles in plants under stress conditions, such as stabilization of membranes and subcellular structures, acting as an ROS scavenger and as a compatible osmolyte that contributes to the conservation of the osmotic gradient in stressed plants [50,51]. In our experimental conditions, upon salt stress, proline content highly increased in control plants, while a more modest increase was observed in EF-treated plants. This may be the consequence of the redox status of these plants, already ameliorated by the upregulation of CAT2 and APX, as well as due to a priming effect exerted by fragment treatments, similar to that induced by proline treatments.

Biostimulants, which are based on molecules or microorganisms that regulate plant physiology and metabolism, may promote plant growth and resilience against abiotic stress, thus representing a very important tool in contemporary agriculture [52,53,54]. A nice example is represented by BALOX^®^, a biostimulant of plant origin that was tested on the responses to salinity of *Lactuca sativa* L. var. longifolia plants exposed to salt concentrations up to 150 mM NaCl; it had a positive effect because it stimulated plant growth and the level of Ca^2+^ and photosynthetic pigments. In addition, it reduced the content of Na^+^ and Cl^−^ in the presence and the absence of salt [55]. In our study, ProSys-derived peptides increased tomato root biomass by up to 18%, shoot growth by up to 9% under salt stress conditions and marketable yield by 50% in open-field experiments. These results reinforce the potential of these peptides as biostimulant tools in agronomic applications. Such characteristics are imperative in varying environments such as those of climate changes that are presently occurring at the global level. Our field trials revealed a direct and proportional enhancement in gross marketable production following treatments with ProSys peptides. These results underline the efficacy of ProSys derivatives as peptide-based biostimulants in promoting yield improvement and economic profitability under adverse climatic conditions. We may speculate that the high fruit production registered in field trials was due to the resilience induced by EFs. In recent years, southern Italy has registered very high temperatures in mid and late summer, the period in which tomatoes are still growing in fields. Such elevated temperatures, besides being a stress *per se*, cause a shortage of water and thus an increase in soil salinity, ultimately damaging the exposed plants and reducing crop yields. Considering that such weather conditions are expected to be present every year due to global warming, the commercialization of novel products that can protect crops, at least in part, from the damage caused by the great heat will likely be able to take advantage of new spaces in the market, as occurred for BALOX^®^. Therefore, once the best formulation process for our EFs is identified, which we are presently working on, with respect to the regulatory rules, their delivery into the market should proceed without major problems.

It is interesting to note that the different seasonal trends of 2023 and 2024 could have affected the effect of peptide’s treatments in different ways, and in fact, in 2023, the production tended to be lower than in 2024; this difference between the productions of the two years could be dictated by the two seasonal trends characterized by different rainfall levels: discontinuous and not high in 2023 and constant in 2024. Furthermore, during 2023, high night-time temperatures combined with irregular rainfall, despite appropriate irrigation management, may have prolonged stress on plants, impacting crop physiology and ultimately fruit development and quality. In fact, in 2023, the production tended to be lower than in 2024. The 2024 season was characterized by more stable and favorable climatic conditions. The rainfall was uniformly distributed throughout the season, and temperatures were slightly lower. These conditions likely maintained a better physiological plant status with a consequent improvement in fruit quality.

In addition to climatic conditions, differences in soil characteristics between the experimental sites may have also contributed to the observed results. In 2023, trials took place in the province of Bologna on a clay–loam soil, which generally offers good water retention [56] but can restrict root aeration under variable humidity caused by rainfall. In 2024, trials were conducted both in the province of Bologna and in the province of Ferrara. In particular, in Ferrara, the soil is predominantly loamy sand, a soil more susceptible to water loss [56], yet under uniform rainfall and well-managed irrigation, it may enhance responsiveness to foliar applications. These pedoclimatic differences, combined with seasonal climatic conditions, likely influenced both the yield and quality of tomato plants.

In the recent past, multiple lines of evidence revealed a prominent role for plant peptides not only in the anti-herbivore and antipathogen responses but also in the modulation of high salinity and drought stress [12]. Indeed, applications of plant peptides prompt plant metabolism through hormone-like effects, which contribute to improving both growth and resistance under salt stress conditions [57]. Different studies have shown that plants treated with plant peptides exhibit higher concentrations of potassium and proline, which are associated with greater tolerance to salt stress. These treatments alleviated the negative impacts of salinity on plant physiology and promoted better growth and yield [58]. Three plant peptides, characterized in Arabidopsis, are known to regulate high salinity and drought stress responses. CLE25, a 12-amino-acid peptide derived from the C-terminal region of the 69-amino-acid CLE25 precursor protein [59], controls stomatal closure under dehydration to prevent water loss by transpiration. Cle25 knockout mutants are more sensitive to dehydration than the wild type. CAPE1, derived from the C-terminus of the 172-amino-acid PROCAPE1, negatively regulates salt tolerance under high salinity [60]. AtPep3, released from a member of the PROPEP gene family, was recently found to enhance the tolerance to high salinity [28]. Both overexpression of AtPROPEP3 and exogenous treatment of synthetic AtPep3 peptide induce salt stress tolerance. Conversely, AtPROPEP3-RNAi lines are hypersensitive under salinity stress, which is recovered by AtPep3 peptide application. The molecular mechanism used by these peptides is largely unknown. In our case, we might speculate that since they are intrinsically disordered, they could possibly establish different and multiple interactions with other proteins, potentially triggering downstream molecular events that lead to metabolic changes associated with stress resilience and growth biostimulation.

This study underscores the potential of peptide-based technologies as innovative agricultural tools to protect crops and enhance their productivity. Such technologies represent an area of increasing and valuable investment for a sustainable farming system.

## 4. Materials and Methods

### 4.1. Laboratory Experiments

#### 4.1.1. Plant Material, Growth Conditions and Plant Treatments

Tomato seeds (cv. ‘San Marzano nano’) were subjected to a two-minute surface sterilization with 70% ethanol and washed for 10 min with 2% sodium hypochlorite, followed by at least five rinses with sterile distilled water. The seeds were then germinated in Petri dishes with wet sterile paper for three days in a growth chamber with a temperature of 24 ± 1 °C and a relative humidity (RH) of 60 ± 5%. Plantlets were then moved to a polystyrene tray with barren sterile S-type substrate (FloraGard; Oldenburg, Germany) once their roots emerged. The growth chamber was set to 26 ± 1 °C, 60 ± 5% RH and 18:6 h light/dark photoperiod. For salt stress experiments, after 2 weeks, plants were transplanted into rhizotrons, transparent square plates (245 × 245 × 25mm, Sarstedt AG & Co. KG, Nümbrecht, Germany), filled with barren sterile S-type substrate (FloraGard; Oldenburg, Germany) under the same growth conditions. All the rhizotrons were wrapped around with aluminum foil and located in order to create a 70° angle with the base as reported by [61]. Plants were irrigated with 120 mL of 100 fM PS1-70, PS1-120 and G1 in PBS buffer 0.1 X as previously described [8]. After 24 h, plants were irrigated with 100 mL of 150 mM NaCl. Controls were irrigated with simple water. Saline treatment was repeated every two days for two weeks. Plants were arranged in a completely randomized block design with eight replicates. Leaf samples from control and treated plants were harvested eight days after salt treatment and used for proline quantification and gene expression analysis.

#### 4.1.2. PS1-70, PS1-120, G1 and Production

Expression and purification of PS1-70 and PS1-120 fragments was carried out as previously described [10]. Briefly, two DNA fragments, ps1-70 and ps1-120, were amplified via polymerase chain reaction starting from the ProSys full-length cDNA as template. The resulting inserts were ligated into a pETM11 t vector, and the plasmids were used to transform E. coli BL21(DE3) cells. Large-scale production of PS1-70 and PS1-120 was carried out as previously described [10]. Pellets were disrupted by sonication on ice, and after centrifugation, the supernatant of each ProSys fragment was purified by an ÄKTA FPLC, on a 1 mL HisTrap FF column (GE Healthcare, Milan, Italy), according to the manufacturer’s instructions (GE Healthcare, Milan, Italy). Eluted fragments were dialyzed in 20 mM Tris–HCl, 50 mM NaCl, 100 mM PMSF, 1 mM DTT, pH 8.0, and purified by size-exclusion chromatography (SEC) on a Superdex 75 10/300 HP (GE Healthcare Milan, Italy), in PBS 1X. The purity level of the recombinant fragments was assessed by SDS-PAGE on a 15% gel using Biorad Precision Plus Protein All Blue Standards (10–250 kDa) as molecular mass markers. LC-ESI-MS analysis of the protein, performed as previously described in [62], confirmed their identities. Once prepared, aliquots were stocked at −20 °C before use. Synthetic G1 was obtained by external services.

#### 4.1.3. Proline Quantification

Proline concentration was quantified with a ninhydrin-based colorimetric test on two technical duplicates for each biological replicate, as previously described [63]. Proline concentration was expressed in µmol g^−1^ fresh weight after comparison with a standard curve.

#### 4.1.4. Molecular Analysis

Tomato leaves harvested from control and salt-irrigated plants, treated or not with PS1-70, PS1-120 and G1, were frozen in liquid nitrogen and stored at −80 °C. RNA extraction, single-stranded cDNA synthesis and real-time RT-PCRs were carried out as previously described [64]. Primers and their main features are described in Supplementary Material Table S3.

#### 4.1.5. Biometric Analysis

Biometric data were collected 15 days after salt treatment to evaluate shoot and root biomass, stomatal density and stomatal area. The plants were cut at the collar and weighed to measure SFW. For root area measurement, one side of the rhizotron was disassembled and photographed. Each image was examined using ImageJ v1.52a (U.S. National Institutes of Health, Bethesda, MD, USA). Stomatal density and area were detected on microscope slides of leaves imprinted with cyanoacrylate [65]. A bright-field microscope (BX60; Olympus Corporation, Tokyo, Japan).) was used to capture 20 photographs through a camera (CAMEDIA C4040,Olympus Corporation, Tokyo, Japan). Stomatal size in terms of length and width (μm) of guard cells was measured on 10 stomata per picture, while stomatal density (number of stomata per mm^2^) was measured on four images per leaf impression.

### 4.2. Open-Field Trials

#### 4.2.1. Experimental Setup and Growth Conditions

Field trials were conducted in 2023 (Experiment 1) and in 2024 (Experiments 2 and 3) on commercial farms located in the provinces of Bologna and Ferrara, in the Emilia-Romagna region of northern Italy. Experiment 1 was conducted in San Giovanni in Persiceto (BO) at the commercial farm “Azienda Agricola Guzzetti Fabio” (latitude: 44°39′4.33″ N; longitude: 11°14′12.23″ E). Experiment 2 was carried out in Lagosanto at “Società Agricola Porto Felloni di Salvagnin L. & C. S. S.” (latitude: 44°44′58.80″ N; longitude: 12°9′41.45″ E), and Experiment 3 in Vedrana, Budrio, at Società Agricola Busato (latitude: 44°33′54.99″ N; longitude: 11°33′24.91″ E).

The tomato crop was selected for all experiments, with specific cultivars differing across trials. In Experiment 1, the variety Barrique was cultivated, while Fokker was used in Experiment 2, and UG 1122713 F1 in Experiment 3. These trials were conducted in areas representative of industrial tomato production, integrated within commercial crop systems that also include Zea mays (maize). Weather conditions during the two growing seasons are reported in Figure 5 and in Table 3, Table 4 and Table 5. The conventional till technique was used for soil tillage. Crop water requirements were completely satisfied by localized drip irrigation (200 L/ha) until 7 days before harvesting. All studies were carried out in accordance with the principles of good experimental practices (GEPs) and EPPO guidelines.

#### 4.2.2. Experimental Design and Plant Treatments

Tomato seedlings were transplanted on 23 May 2023 for Experiment 1, on 20 May 2024 for Experiments 2, and on 9 May 2024 for Experiment 3, into 20 m^2^ plots (width: 4 m, length: 5 m) with 240 plants per thesis. The experimental design followed a randomized complete block (RCB) with four replicates.

In Experiment 1, tomato plants were treated with 100 fM solutions of the experimental fragments G1 and PS1-70 via foliar application using a Honda WJR backpack sprayer. Treatments were applied three times: on 9 June 2023 (15 days post-transplant), 11 July 2023 (44 days post-transplant) and 10 August 2023 (53 days post-transplant). For Experiment 2, plants were treated with 100 fM solutions of G1 and PS1-70 using the same sprayer, testing two timelines of applications: Experiment 2a, 5 June 2024 (15 days post-transplant), 4 July 2024 (30 days after the first application) and 5 August 2024 (60 days after the first application), and 2b, 5 June 2024 (15 days post-transplant), 27 June 2024 (20 days after the first application) and 18 July 2024 (40 days after the first application). The main difference between Experiments 2a and 2b lies in the frequency of treatments: following the initial application, plants were treated every 30 days in 2a and every 20 days in timeline 2b. In Experiment 3, 100 fM solutions of G1 and PS1-70 were applied three times: on 17 June 2024 (3.0 days post-transplant), 4 July 2024 (50 days post-transplant) and 25 July 2024 (70 days post-transplant), using the Honda WJR backpack sprayer. A summary of transplanting and peptide treatment intervals is reported in Table 6.

#### 4.2.3. Plant Harvesting and Quality Analysis

Tomato fruits were harvested on 28 August 2023, 27 August 2024 and 13 August 2024 for Experiments 1, 2 and 3, respectively, and subsequently sorted into two groups: ripe fruits and immature or rotten fruits. Both groups were weighed and counted. The weight (kg) and number of ripe fruits were used to calculate the marketable yield, expressed in tons per hectare (t/ha). The values of GMP were obtained considering a price of 140 EUR/ton, attributable to products with a °Brix level between 4.8 and 5.2.

Two hundred tomato fruits per thesis were harvested at a late stage of ripening (full red color) for °Brix analysis of the 2023 and 2024 trials. Fruits were squeezed, and one to two drops of clear juice were placed on the prism of an ocular refractometer previously calibrated with distilled water with a range of 0 to 32 °Brix, a resolution of 0.2 °Brix and a compensated temperature. Between samples, the prism of the refractometer was washed with distilled water and dried before use. The results obtained were multiplied by the dilution factor (water and pulp) and expressed in °Brix. Detailed values of marketable and total production, GMP and °Brix for each replicate per treatment across all three experiments are provided in Supplementary Material Tables S4–S19.

### 4.3. Statistical Analysis

Gene expression analysis and biometric measurements were analyzed using the Student’s *t*-test (*p* < 0.05) or two-way ANOVA procedure with Tukey’s or Duncan’s post hoc test (*p* < 0.05). The results of post hoc pairwise comparisons were expressed as a form of compact letter display (CLD). Overlapping letters are nonsignificant (*p* > 0.05), while separate letter classes indicate *p* < 0.05 or better. The ANOVA was performed using SPSS (Statistical Package for Social Sciences) v21 software (IBM, Armonk, NY, USA) 6, version 23. Total and marketable production (T/ha) results from open-field trials were analyzed using Levene’s test to verify homogeneity of variances. The Shapiro–Wilk test and a kurtosis test were used to evaluate data normality. Means were compared using the Student–Newman–Keuls (SNK) test with a significance threshold of *p* ≤ 0.05. The coefficient of variation (CV) was calculated to assess the relative dispersion of the data.

For open-field trials, a single replication is an individual field plot, while a “randomized block” is the random arrangement, within the open-field trial, of the replica plots of each thesis (4 theses for Experiment 1 and 3 theses for the others). Each thesis is made up of 240 plants; consequently, having 4 replications, each plot has 60 plants.

## 5. Conclusions

The biostimulant activity of EFs in promoting biomass growth, even under salinity conditions, is a very interesting characteristic that highlights the great potential of the novel fragments used in this study in agriculture. Intriguingly, these results were obtained in open-field experiments, which makes the data much more reliable than those obtained only in controlled laboratory conditions. The treatments were shown to positively affect tomato plants in terms of both yield (total and marketable) and quality (°Brix), highlighting the great efficacy of these bio-inspired tools. Thus, their integration into crop management strategies may represent an innovative approach to mitigate the impacts of climate variability on agricultural production in a sustainable way. Notably, these peptides are active at extremely low concentrations (in the femtomolar range), which translates into very low application costs, making them economically attractive for large-scale use. Currently, we are developing and testing suitable formulations to support their future commercialization in the sustainable agriculture market.

## 6. Patents

The protein fragments (PS1-70 and PS 1-120) and G1 are included in the patent file number WO 2022/024015 A1.

## Figures and Tables

**Table 1:** Shoot fresh weight (SFW) and root area of tomato plants treated with PS1-70, PS1-120 and G1 fragments in the absence (0 mM NaCl) and presence (150 mM NaCl) of salt. Error bars indicate standard error (*n* = 8). Statistical analysis was performed with two-way ANOVA (* = *p* < 0.05; *** = *p* < 0.001; ns = not significant). For statistically significant differences induced by one of the two factors, different letters indicate significant differences according to the Duncan post hoc test (*p* < 0.05).

Treatment	SFW (g)	Root Area (cm^2^)
Salt (S)		
0 mM NaCl	28.4 a	102.1 a
150 mM NaCl	21.4 b	82.8 b
EFs		
Control	23.6 b	86.7 a
PS1-70	25.8 a	92.1 a
PS1-120	24.3 ab	95.8 a
G1	25.7 a	93.5 a
Interaction		
S	***	***
EFs	*	ns
S × EFs	ns	***

**Table 2:** Gross marketable production (GMP) of plants treated with 100 fM solutions of PS1-70 and G1 in the 2024 trial (Lagosanto, Ferrara). The values are mean ± standard error (n = 4). Means were compared using the Student–Newman–Keuls (SNK) test (*p* ≤ 0.05). Letters indicate statistically significant differences between the experimental groups.

Trial	Treatments	GMP (EUR/ha)
Lagosanto (Ferrara, 2024)	Farm line	9310.0 d
	PS1-70	14,396.7 bc
	G1	15,843.3 abc

**Table 3:** Daily temperatures, relative humidity, rain events, leaf wetness and wind during 2023 open-field trial (Experiment 1) in San Giovanni in Persiceto (BO).

				Temperature °C			Relative Humidity %			mm/m^2^	Minute	m/s
S	M	Y	Date	TMx	TAv	TMn	RH Mx	RH Av	RH Mn	Rainfall	Leaf Wetness	Wind
21	5	2023	23 May 2023	29.6	21.1	12.2	100	77	39	0.0	540	1.1
21	5	2023	24 May 2023	27.3	19.7	13.3	100	87	51	0.0	480	1.2
21	5	2023	25 May 2023	25.8	19.9	16.0	100	93	68	0.0	480	1.5
22	5	2023	26 May 2023	29.4	21.2	12.1	100	78	42	0.0	540	1.4
22	5	2023	27 May 2023	27.4	21.0	15.8	100	81	45	6.0	540	1.3
22	5	2023	28 May 2023	27.8	20.9	12.2	100	73	41	0.0	420	1.9
22	5	2023	29 May 2023	28.3	20.5	12.4	100	76	39	0.0	600	1.3
22	5	2023	30 May 2023	27.8	21.0	14.2	100	73	41	0.0	360	1.2
22	5	2023	31 May 2023	26.8	20.8	14.7	100	74	42	0.0	300	1.9
22	6	2023	1 June 2023	27.9	20.6	12.7	100	75	43	0.0	420	1.8
23	6	2023	2 June 2023	29.8	21.8	14.2	100	73	36	0.0	180	0.9
23	6	2023	3 June 2023	27.8	19.3	14.8	100	90	52	7.6	420	0.9
23	6	2023	4 June 2023	26.3	19.3	13.7	100	93	56	6.4	840	1.5
23	6	2023	5 June 2023	21.1	17.9	16.7	100	100	99	21.2	1020	1.4
23	6	2023	6 June 2023	27.0	20.5	16.5	100	90	53	0.0	600	1.1
23	6	2023	7 June 2023	26.5	20.1	14.4	100	95	65	1.2	720	1.3
23	6	2023	8 June 2023	28.6	21.0	13.9	100	83	39	0.0	600	1
24	6	2023	9 June 2023	29.6	22.2	14.7	100	80	43	0.0	840	1.1
24	6	2023	10 June 2023	24.8	21.5	18.3	100	97	76	4.2	720	0.8
24	6	2023	11 June 2023	28.9	22.3	15.1	100	82	48	0.0	540	1.5
24	6	2023	12 June 2023	28.4	23.2	17.4	100	78	45	0.0	360	1.4
24	6	2023	13 June 2023	26.2	20.1	15.8	100	93	57	0.0	660	2
24	6	2023	14 June 2023	26.5	20.2	14.6	100	88	55	0.6	660	2.3
24	6	2023	15 June 2023	28.1	21.5	14.5	100	75	39	0.0	540	1.6
25	6	2023	16 June 2023	28.9	22.0	14.0	100	75	38	0.0	420	1.4
25	6	2023	17 June 2023	30.4	22.6	14.0	100	72	21	7.6	420	1.5
25	6	2023	18 June 2023	31.1	23.1	13.9	100	75	38	0.0	600	1.1
25	6	2023	19 June 2023	32.1	24.6	17.0	100	69	36	0.0	60	0.8
25	6	2023	20 June 2023	32.2	25.0	16.8	100	60	35	0.0	0	0.9
25	6	2023	21 June 2023	33.1	25.7	17.9	100	72	39	0.0	60	1
25	6	2023	22 June 2023	35.0	27.4	20.2	100	74	39	0.0	180	1
26	6	2023	23 June 2023	31.1	24.8	19.0	100	77	51	0.0	120	1.2
26	6	2023	24 June 2023	30.7	24.4	15.8	100	70	33	0.0	480	1.6
26	6	2023	25 June 2023	30.7	23.8	15.3	100	66	32	0.0	420	1.7
26	6	2023	26 June 2023	34.0	25.1	14.6	100	65	28	0.0	360	1.7
26	6	2023	27 June 2023	34.9	26.5	17.4	100	71	38	8.4	480	1.8
26	6	2023	28 June 2023	26.9	22.7	19.9	100	92	66	0.0	240	1.3
26	6	2023	29 June 2023	30.2	24.4	16.9	100	66	33	0.0	60	2.7
27	6	2023	30 June 2023	25.4	21.5	18.7	100	89	58	1.2	360	2.8
27	7	2023	1 July 2023	28.0	22.1	17.4	100	85	53	0.0	420	1.7
27	7	2023	2 July 2023	32.6	25.0	16.5	100	75	41	0.0	420	1.2
27	7	2023	3 July 2023	32.7	24.2	18.5	100	84	44	29.4	480	1.6
27	7	2023	4 July 2023	30.3	23.9	17.0	100	79	44	1.0	240	1.6
27	7	2023	5 July 2023	31.0	24.4	17.1	100	79	48	0.0	900	2.6
27	7	2023	6 July 2023	26.3	22.5	17.5	100	90	66	0.0	1140	1
28	7	2023	7 July 2023	30.8	23.8	15.8	100	75	42	0.0	900	2.1
28	7	2023	8 July 2023	32.3	24.8	14.9	100	68	34	0.0	660	1.7
28	7	2023	9 July 2023	34.4	27.1	18.2	100	69	36	0.0	540	2.3
28	7	2023	10 July 2023	36.6	28.6	20.2	100	71	37	0.0	480	1.1
28	7	2023	11 July 2023	36.5	29.4	21.3	100	67	38	0.0	360	1.5
28	7	2023	12 July 2023	34.8	28.3	22.2	100	78	50	0.0	1140	1
28	7	2023	13 July 2023	30.3	25.8	23.0	100	92	68	0.0	1020	2
29	7	2023	14 July 2023	32.7	26.8	21.7	100	82	51	0.0	600	1.5
29	7	2023	15 July 2023	34.6	27.3	19.5	100	72	38	0.0	420	2.1
29	7	2023	16 July 2023	36.8	28.6	19.5	100	69	33	0.0	420	1.5
29	7	2023	17 July 2023	36.2	28.7	19.2	100	64	37	0.0	180	1
29	7	2023	18 July 2023	36.8	28.8	20.0	100	75	45	0.0	360	0.9
29	7	2023	19 July 2023	36.5	29.3	22.4	100	79	41	0.0	360	1.8
29	7	2023	20 July 2023	33.7	27.1	19.5	100	78	49	0.0	300	1.8
30	7	2023	21 July 2023	32.9	25.9	18.7	100	82	42	0.0	480	1.9
30	7	2023	22 July 2023	31.7	24.9	20.3	100	92	55	6.0	660	2.3
30	7	2023	23 July 2023	33.5	25.2	16.6	100	81	43	0.0	540	1.6
30	7	2023	24 July 2023	32.8	24.9	17.0	100	87	46	0.0	420	1.2
30	7	2023	25 July 2023	31.3	24.9	20.0	100	79	25	0.0	60	2.2
30	7	2023	26 July 2023	30.0	23.1	16.6	100	72	37	0.6	240	2.2
30	7	2023	27 July 2023	30.5	22.9	14.6	100	74	34	0.0	300	1.8
31	7	2023	28 July 2023	31.0	23.3	15.5	100	72	38	0.0	120	2.4
31	7	2023	29 July 2023	33.6	25.9	17.0	100	72	42	0.0	240	0.9
31	7	2023	30 July 2023	33.2	27.1	20.3	100	70	43	0.0	960	1.2
31	7	2023	31 July 2023	32.9	26.2	19.7	100	79	45	0.0	960	1.5
31	8	2023	1 August 2023	33.1	25.3	18.6	100	72	34	0.0	960	1.6
31	8	2023	2 August 2023	33.7	25.2	16.3	100	70	39	0.0	1080	1.6
31	8	2023	3 August 2023	34.5	27.1	19.1	100	64	40	0.0	1020	2
32	8	2023	4 August 2023	26.5	23.2	17.8	100	66	39	0.8	1080	1.7
32	8	2023	5 August 2023	22.4	18.4	16.1	100	98	74	13.8	1080	1.9
32	8	2023	6 August 2023	30.4	22.0	14.1	100	66	20	0.0	420	2.6
32	8	2023	7 August 2023	28.3	20.4	13.7	100	64	23	0.0	480	2.5
32	8	2023	8 August 2023	30.0	20.7	10.7	100	58	22	0.0	60	2.2
32	8	2023	9 August 2023	31.0	22.6	14.7	83	58	28	0.0	420	1.2
32	8	2023	10 August 2023	31.0	23.7	15.3	100	73	41	0.0	540	1.6
33	8	2023	11 August 2023	31.8	25.0	16.7	100	68	42	0.0	480	1.9
33	8	2023	12 August 2023	33.1	25.1	15.8	100	70	39	0.0	480	1.9
33	8	2023	13 August 2023	33.4	26.5	18.1	100	71	45	0.0	420	1.9
33	8	2023	14 August 2023	34.1	26.3	16.5	100	70	33	0.0	480	1.4
33	8	2023	15 August 2023	34.2	26.3	17.3	100	70	38	0.0	420	1.8
33	8	2023	16 August 2023	34.1	27.0	18.6	100	70	37	0.0	480	1.3
33	8	2023	17 August 2023	33.1	26.1	18.9	100	78	44	0.0	600	1.3
34	8	2023	18 August 2023	34.2	26.9	18.6	100	72	41	0.0	420	1
34	8	2023	19 August 2023	35.3	27.7	19.3	100	70	38	0.0	420	1.3
34	8	2023	20 August 2023	35.0	27.4	19.1	100	70	38	0.0	420	1.1
34	8	2023	21 August 2023	35.5	28.1	20.8	100	65	36	0.0	240	1.3
34	8	2023	22 August 2023	36.6	28.2	19.3	100	66	31	0.0	420	1.2
34	8	2023	23 August 2023	37.9	28.8	18.9	100	62	26	0.0	420	1.4
34	8	2023	24 August 2023	38.8	29.2	18.9	100	59	23	0.0	360	1.3
35	8	2023	25 August 2023	38.5	29.7	20.6	96	56	30	0.0	420	1.8
35	8	2023	26 August 2023	37.2	27.7	18.6	100	57	27	0.0	180	1.1
35	8	2023	27 August 2023	30.4	25.2	20.0	100	69	47	0.0	240	1.2
35	8	2023	28 August 2023	28.9	23.0	17.6	100	80	43	1.0	720	1.9
35	8	2023	29 August 2023	20.0	17.5	15.5	100	100	100	10.8	1200	3.5
35	8	2023	30 August 2023	25.0	20.5	15.6	100	77	50	0.2	480	1.4
35	8	2023	31 August 2023	27.9	20.4	13.6	100	80	44	0.0	540	1.6
36	9	2023	1 September 2023	29.1	22.0	14.5	100	74	43	0.0	120	1.3
36	9	2023	2 September 2023	30.8	23.4	15.9	100	76	41	0.0	300	1.4
36	9	2023	3 September 2023	31.6	24.3	17.3	100	75	39	0.0	180	1.5
36	9	2023	4 September 2023	29.3	23.8	17.9	100	71	44	0.0	600	1.6
36	9	2023	5 September 2023	26.8	22.3	16.1	81	58	38	0.0	480	3.2
36	9	2023	6 September 2023	29.2	21.1	13.6	100	66	40	0.0	600	2.2
36	9	2023	7 September 2023	29.9	22.1	15.4	100	69	37	0.0	600	1.1
37	9	2023	8 September 2023	30.5	22.5	14.7	100	64	36	0.0	600	1.3
37	9	2023	9 September 2023	30.8	22.7	14.7	100	66	32	0.0	540	1.2
37	9	2023	10 September 2023	30.9	22.6	14.8	99	64	36	0.0	480	1.1
37	9	2023	11 September 2023	31.6	23.3	14.2	100	65	33	0.0	360	0.8
37	9	2023	12 September 2023	33.0	24.1	14.3	100	64	29	0.0	420	1.8
37	9	2023	13 September 2023	29.8	23.2	17.0	100	69	37	0.0	360	1.5
37	9	2023	14 September 2023	29.5	22.5	16.2	100	80	49	0.0	600	1.7
38	9	2023	15 September 2023	26.8	21.4	18.6	100	94	61	8.4	1020	1.5
38	9	2023	16 September 2023	26.6	20.7	15.9	100	99	87	0.6	780	1.4
38	9	2023	17 September 2023	28.5	22.7	18.4	100	92	60	0.2	720	1.5
38	9	2023	18 September 2023	26.3	22.2	18.5	100	97	77	0.0	480	1
38	9	2023	19 September 2023	29.7	22.7	16.2	100	87	54	3.4	420	1.4
38	9	2023	20 September 2023	25.2	20.2	15.1	100	94	73	0.0	240	1.1
38	9	2023	21 September 2023	27.8	22.0	17.4	100	84	51	0.4	480	1.5
39	9	2023	22 September 2023	25.0	21.6	17.2	100	88	52	0.0	60	1.5
39	9	2023	23 September 2023	26.4	19.6	12.8	100	75	39	10.2	420	2
39	9	2023	24 September 2023	24.0	17.5	12.5	100	81	43	0.2	480	1.7
39	9	2023	25 September 2023	25.7	17.7	10.5	100	82	42	0.0	480	1.9
39	9	2023	26 September 2023	26.8	19.0	12.4	100	85	52	0.0	60	1
39	9	2023	27 September 2023	27.9	19.8	13.6	100	82	41	0.0	360	1.3
39	9	2023	28 September 2023	28.1	19.6	12.4	100	77	39	0.0	240	1.3
40	9	2023	29 September 2023	28.4	19.6	12.6	100	75	37	0.0	420	1
40	9	2023	30 September 2023	28.5	19.8	12.1	100	72	32	0.0	240	0.8

S: week. M: month. TMx: maximum daily temperature, °C. TAv: average daily temperature, °C. TMn: minimum daily temperature, °C. RH Mx: maximum daily relative humidity, %. RH Av: average daily relative humidity, %. RH Mn: minimum daily relative humidity, %. Rainfall: daily rainfall, mm/m^2.^ Leaf Wetness: minute daily leaf wetness. Wind: average daily wind, m/s.

**Table 4:** Daily temperatures, relative humidity, rain events, leaf wetness and wind during 2024 open-field trials (Experiment 2a and 2b) in Lagosanto (FE).

				Temperature °C			Relative Humidity %			mm/m^2^	Minute	m/s
S	M	Y	Date	TMx	TAv	TMn	RH Mx	RH Av	RH Mn	Rainfall	Leaf Wetness	Wind
18	5	2024	1 May 2024	17.8	14.6	11.8	100	96	0	10.8	1075	1.3
18	5	2024	2 May 2024	18.9	15.2	11.3	100	83	0	1.0	660	2.4
18	5	2024	3 May 2024	19.1	14.3	10.9	100	83	0	1.0	725	1.1
18	5	2024	4 May 2024	20.8	14.4	6.0	100	81	0	0.0	630	0.8
19	5	2024	5 May 2024	22.5	14.8	6.1	100	75	0	0.0	490	0.9
19	5	2024	6 May 2024	22.7	16.1	8.0	99	74	0	0.0	255	2
19	5	2024	7 May 2024	21.6	16.6	12.5	100	88	55	7.0	670	1.7
19	5	2024	8 May 2024	23.7	16.2	9.3	100	86	49	0.2	550	1.2
19	5	2024	9 May 2024	26.4	19.5	12.3	100	69	68	0.0	300	2.5
19	5	2024	10 May 2024	26.6	19.4	11.8	87	60	58	0.0	0	2.6
19	5	2024	11 May 2024	27.8	18.5	8.9	94	63	60	0.0	0	1.5
20	5	2024	12 May 2024	25.0	17.8	9.0	100	76	53	0.0	405	0.9
20	5	2024	13 May 2024	24.6	17.8	10.3	100	82	60	0.0	585	1.1
20	5	2024	14 May 2024	25.9	18.0	9.5	100	79	53	0.0	460	1
20	5	2024	15 May 2024	26.2	19.5	11.8	100	78	56	0.0	480	2.1
20	5	2024	16 May 2024	25.1	19.0	14.0	100	84	56	0.0	550	1.2
20	5	2024	17 May 2024	24.6	18.4	10.8	100	77	50	0.0	625	1.8
20	5	2024	18 May 2024	27.0	18.5	9.7	100	72	50	0.2	490	1.3
21	5	2024	19 May 2024	25.0	17.8	11.5	100	85	56	0.0	670	1.2
21	5	2024	20 May 2024	26.2	19.2	13.4	100	88	72	17.8	805	1.3
21	5	2024	21 May 2024	26.1	19.9	14.8	100	83	40	10.0	775	1.2
21	5	2024	22 May 2024	25.7	18.9	10.8	100	74	67	0.2	450	1.3
21	5	2024	23 May 2024	33.8	19.9	10.5	100	61	35	1.0	0	1.2
21	5	2024	24 May 2024	35.2	22.9	12.3	57	47	33	0.0	0	0.9
21	5	2024	25 May 2024	31.5	21.0	15.0	66	56	44	0.0	0	0.8
22	5	2024	26 May 2024	37.7	24.4	11.3	72	53	40	0.0	0	0.9
22	5	2024	27 May 2024	29.6	22.0	13.5	88	52	38	0.8	0	0.7
22	5	2024	28 May 2024	24.6	23.8	22.7	70	59	40	0.0	0	0.6
22	5	2024	29 May 2024	25.6	24.2	22.8	63	59	50	0.0	0	0.5
22	5	2024	30 May 2024	25.9	24.1	21.2	62	58	51	0.0	0	0.4
22	5	2024	31 May 2024	25.1	24.0	23.1	63	59	50	0.0	0	0.4
22	6	2024	1 June 2024	25.4	24.1	22.1	58	51	40	0.0	0	0.9
23	6	2024	2 June 2024	25.2	24.2	23.0	54	51	43	0.0	0	0.9
23	6	2024	3 June 2024	25.6	24.0	22.5	60	54	49	0.4	0	0.8
23	6	2024	4 June 2024	31.3	25.0	21.0	55	49	40	2.6	0	0.9
23	6	2024	5 June 2024	32.6	22.8	16.6	89	63	41	2.2	0	2
23	6	2024	6 June 2024	28.1	23.0	15.5	96	74	42	0.0	0	1.7
23	6	2024	7 June 2024	32.3	24.9	18.1	96	71	51	0.0	0	2.1
23	6	2024	8 June 2024	31.9	25.1	18.8	98	72	48	0.0	0	2
24	6	2024	9 June 2024	31.2	25.0	20.4	100	76	39	0.0	65	1.7
24	6	2024	10 June 2024	30.1	23.9	17.9	100	76	42	6.0	495	1.6
24	6	2024	11 June 2024	28.3	22.7	16.5	100	68	40	0.2	155	1.4
24	6	2024	12 June 2024	29.1	21.5	16.6	100	74	49	45.4	475	1.2
24	6	2024	13 June 2024	25.5	19.0	15.2	100	82	60	0.2	520	1.1
24	6	2024	14 June 2024	25.0	20.3	12.9	100	74	38	0.0	450	1.8
24	6	2024	15 June 2024	27.6	21.8	14.1	100	74	47	0.0	140	2.4
25	6	2024	16 June 2024	30.8	23.6	16.0	90	59	20	0.0	0	1.6
25	6	2024	17 June 2024	31.3	23.2	14.8	100	67	65	0.0	0	1.9
25	6	2024	18 June 2024	29.6	24.2	17.0	100	77	61	0.0	505	2.2
25	6	2024	19 June 2024	34.2	26.8	20.3	100	74	55	0.0	495	1.6
25	6	2024	20 June 2024	32.3	26.1	20.7	100	76	54	0.0	0	1.2
25	6	2024	21 June 2024	31.1	25.1	21.2	100	88	55	0.0	450	1.6
25	6	2024	22 June 2024	33.0	25.1	17.6	89	62	41	0.0	0	1.5
26	6	2024	23 June 2024	23.4	20.4	16.9	100	88	40	1.2	395	0.7
26	6	2024	24 June 2024	27.7	21.6	18.0	100	89	50	9.8	920	1
26	6	2024	25 June 2024	29.2	21.8	18.3	100	91	50	5.8	745	1.1
26	6	2024	26 June 2024	29.0	21.6	17.5	100	87	56	0.4	600	0.8
26	6	2024	27 June 2024	33.7	24.8	16.4	100	75	47	0.0	295	1.1
26	6	2024	28 June 2024	33.7	26.3	19.5	100	78	63	0.0	130	1.8
26	6	2024	29 June 2024	31.4	26.7	21.0	100	83	59	0.0	460	2.2
27	6	2024	30 June 2024	34.1	27.9	21.5	100	66	40	0.0	225	1.6
27	7	2024	1 July 2024	32.2	25.0	18.2	100	73	48	14.4	255	1.7
27	7	2024	2 July 2024	28.9	23.1	16.4	100	76	40	0.2	575	2.7
27	7	2024	3 July 2024	25.0	21.2	16.2	100	81	51	50.6	350	2.1
27	7	2024	4 July 2024	28.7	21.8	16.1	100	78	57	6.0	610	1.2
27	7	2024	5 July 2024	28.4	23.2	15.8	100	77	50	0.0	445	2
27	7	2024	6 July 2024	31.6	25.6	19.1	100	78	52	0.0	365	2.1
28	7	2024	7 July 2024	30.6	25.8	20.9	100	70	69	0.0	140	1.7
28	7	2024	8 July 2024	32.5	26.9	21.4	100	80	47	0.0	470	1.8
28	7	2024	9 July 2024	36.2	28.1	20.1	100	73	58	0.0	355	1
28	7	2024	10 July 2024	37.7	29.0	21.0	100	76	40	0.0	210	1
28	7	2024	11 July 2024	37.5	29.5	22.9	100	71	42	0.0	0	1.5
28	7	2024	12 July 2024	36.9	29.4	21.9	100	68	52	0.0	30	1.4
28	7	2024	13 July 2024	35.4	28.1	22.4	100	79	45	0.0	460	1.4
29	7	2024	14 July 2024	36.0	27.5	19.6	100	62	50	0.0	0	1.2
29	7	2024	15 July 2024	33.6	27.2	19.9	100	74		0.0	425	1.8
29	7	2024	16 July 2024	35.9	28.1	20.4	100	79	62	0.0	420	1.5
29	7	2024	17 July 2024	34.8	28.1	20.1	100	75	45	0.0	260	1.9
29	7	2024	18 July 2024	34.7	28.2	20.8	100	79		0.0	600	1.7
29	7	2024	19 July 2024	36.8	29.2	21.9	100	73	51	0.0	415	1.1
29	7	2024	20 July 2024	31.6	26.2	20.3	100	79	54	6.0	340	1.7
30	7	2024	21 July 2024	31.3	26.7	20.2	100	82	69	0.0	300	1.9
30	7	2024	22 July 2024	33.2	25.9	22.5	100	86	38	0.8	705	1.3
30	7	2024	23 July 2024	32.6	26.8	21.3	100	79	31	0.2	430	1.5
30	7	2024	24 July 2024	33.4	27.2	20.6	100	77	49	0.0	410	1.3
30	7	2024	25 July 2024	30.3	26.3	21.1	100	75	57	0.0	65	2.3
30	7	2024	26 July 2024	31.0	26.5	20.0	100	70	53	0.0	0	2.2
30	7	2024	27 July 2024	31.5	27.0	21.5	100	79	49	0.0	265	2
31	7	2024	28 July 2024	36.1	28.3	21.2	100	82	55	0.0	560	1.6
31	7	2024	29 July 2024	34.2	28.0	22.8	100	79	57	0.0	235	1.3
31	7	2024	30 July 2024	31.8	26.3	20.4	100	74	49	0.0	425	2
31	7	2024	31 July 2024	35.0	27.3	20.2	100	76	46	0.0	430	1.7
31	8	2024	1 August 2024	34.1	28.2	22.2	100	81	60	0.8	595	2.1
31	8	2024	2 August 2024	31.4	26.2	20.5	100	87	43	0.0	310	1.7
31	8	2024	3 August 2024	29.9	24.2	21.0	100	94	52	11.2	1085	1.1
32	8	2024	4 August 2024	33.0	26.3	20.2	100	79	52	0.2	515	1.3
32	8	2024	5 August 2024	30.8	26.4	21.6	100	80	47	0.0	270	1.9
32	8	2024	6 August 2024	32.1	26.4	21.0	100	81	41	0.0	415	1.6
32	8	2024	7 August 2024	33.8	26.5	20.2	100	83	48	10.6	595	1.8
32	8	2024	8 August 2024	33.8	25.4	19.0	100	84	37	0.0	640	1.1
32	8	2024	9 August 2024	33.9	27.0	20.0	100	81	43	0.2	490	1.7
32	8	2024	10 August 2024	33.4	27.9	21.4	100	76	55	0.0	540	1.6
33	8	2024	11 August 2024	36.2	28.2	20.8	100	76	50	0.0	550	1.6
33	8	2024	12 August 2024	36.6	28.6	20.9	100	76	48	0.0	480	1.7
33	8	2024	13 August 2024	34.7	28.7	21.8	100	79	52	0.0	450	1.8
33	8	2024	14 August 2024	35.8	28.6	22.7	100	74	40	0.0	275	1.6
33	8	2024	15 August 2024	34.3	26.7	19.9	100	76	65	0.0	185	0.9
33	8	2024	16 August 2024	36.2	27.9	21.4	100	74	54	0.0	65	1
33	8	2024	17 August 2024	36.6	26.7	21.2	100	79	54	10.0	365	1.1
34	8	2024	18 August 2024	32.1	24.8	20.4	100	84	61	0.0	640	1.1
34	8	2024	19 August 2024	25.9	21.5	19.5	100	100	50	18.8	1425	0.8
34	8	2024	20 August 2024	29.7	24.5	20.9	100	90	53	2.6	750	1.2
34	8	2024	21 August 2024	33.6	26.5	20.4	100	80	48	0.0	535	1
34	8	2024	22 August 2024	30.6	26.2	21.3	100	80	44	0.0	295	1.8
34	8	2024	23 August 2024	31.8	26.3	20.5	100	83	43	0.0	510	1.7
34	8	2024	24 August 2024	34.5	27.4	21.9	100	84	41	0.0	635	1.7
35	8	2024	25 August 2024	35.7	26.9	20.0	100	80	38	0.2	600	1.2
35	8	2024	26 August 2024	33.8	26.7	20.7	100	82	45	0.2	545	1.3
35	8	2024	27 August 2024	31.7	23.2	20.6	100	94	36	0.0	415	0.5

S: week. M: month. TMx: maximum daily temperature, °C. TAv: average daily temperature, °C. TMn: minimum daily temperature, °C. RH Mx: maximum daily relative humidity, %. RH Av: average daily relative humidity, %. RH Mn: minimum daily relative humidity, %. Rainfall: daily rainfall, mm/m^2.^ Leaf Wetness: minute daily leaf wetness. Wind: average daily wind, m/s.

**Table 5:** Daily temperatures, relative humidity, rain events, leaf wetness and wind during 2024 open-field trial (Experiment 3) in Vedrana, Budrio (BO).

				Temperature °C			Relative Humidity %			mm/m^2^	Minute	m/s
S	M	Y	Date	TMx	TAv	TMn	RH Mx	RH Av	RH Mn	Rainfall	Leaf Wetness	Wind
18	5	2024	1 May 2024	25.4	17.3	8.2	100	92	70	0.0	660	1.6
18	5	2024	2 May 2024	21.0	15.9	10.0	100	79	53	2.8	420	1.3
18	5	2024	3 May 2024	19.7	15.0	9.6	100	73	39	0.4	240	2.5
18	5	2024	4 May 2024	21.8	14.7	5.6	100	79	53	0.2	300	1.7
19	5	2024	5 May 2024	23.4	16.8	12.5	96	65	38	2.0	0	1.3
19	5	2024	6 May 2024	26.0	18.4	11.0	97	67	41	0.0	120	1.3
19	5	2024	7 May 2024	22.6	17.4	12.9	100	94	80	0.0	960	1.1
19	5	2024	8 May 2024	18.0	14.5	11.5	100	88	55	8.8	600	1.1
19	5	2024	9 May 2024	22.2	15.9	11.3	100	81	49	2.0	180	1.3
19	5	2024	10 May 2024	24.2	17.4	11.3	100	71	35	0.0	360	1.2
19	5	2024	11 May 2024	25.0	17.3	9.7	96	64	36	0.0	0	1.3
20	5	2024	12 May 2024	27.5	18.4	9.0	96	67	35	0.0	60	1.2
20	5	2024	13 May 2024	27.1	19.3	11.4	100	79	47	0.0	360	1.8
20	5	2024	14 May 2024	25.2	18.8	12.2	100	83	48	12.4	420	1.5
20	5	2024	15 May 2024	24.9	19.1	14.2	100	95	77	0.0	840	2
20	5	2024	16 May 2024	25.1	20.1	15.0	100	95	78	0.0	120	2.3
20	5	2024	17 May 2024	25.0	20.1	15.2	99	61	34	0.0	60	1.6
20	5	2024	18 May 2024	25.2	19.7	14.2	100	74	45	2.0	240	1.2
21	5	2024	19 May 2024	23.0	19.5	16.0	100	81	41	0.0	420	1.8
21	5	2024	20 May 2024	22.0	18.5	15.0	100	94	74	0.0	960	1.5
21	5	2024	21 May 2024	24.5	19.9	15.3	100	85	54	17.4	720	1.9
21	5	2024	22 May 2024	25.1	20.6	16.1	100	75	44	1.2	360	1.8
21	5	2024	23 May 2024	27.0	21.1	15.1	100	77	44	0.0	540	1.3
21	5	2024	24 May 2024	25.0	20.5	16.0	100	82	51	1.8	540	1.5
21	5	2024	25 May 2024	23.0	19.5	16.0	100	89	65	12.6	300	1.6
22	5	2024	26 May 2024	24.0	19.0	14.0	100	77	44	1.4	420	1.4
22	5	2024	27 May 2024	22.3	18.8	15.3	100	78	45	0.0	360	1.4
22	5	2024	28 May 2024	24.0	19.0	14.0	100	86	62	0.0	120	1.6
22	5	2024	29 May 2024	21.0	18.0	15.0	100	82	51	0.0	480	1.4
22	5	2024	30 May 2024	26.0	20.2	14.1	100	78	49	0.0	480	1.2
22	5	2024	31 May 2024	26.1	20.1	14.0	100	84	63	2.6	420	1.4
22	6	2024	1 June 2024	22.2	16.5	11.7	94	67	31	8.8	0	1.6
23	6	2024	2 June 2024	26.9	19.4	9.5	100	79	49	0.0	180	1.5
23	6	2024	3 June 2024	23.8	18.2	13.7	100	76	50	0.0	180	1.7
23	6	2024	4 June 2024	25.2	18.8	13.7	98	77	40	1.2	180	1.5
23	6	2024	5 June 2024	27.1	20.3	14.3	100	71	36	0.0	300	1.3
23	6	2024	6 June 2024	29.1	22.0	14.4	97	69	41	0.0	0	1.5
23	6	2024	7 June 2024	30.5	23.2	15.7	92	67	44	0.0	0	1.4
23	6	2024	8 June 2024	31.7	24.7	17.5	87	61	43	0.0	0	1.3
24	6	2024	9 June 2024	31.8	25.3	18.7	94	73	57	0.0	0	1.3
24	6	2024	10 June 2024	27.3	23.1	19.4	100	69	44	0.4	300	1.2
24	6	2024	11 June 2024	28.7	23.4	18.6	78	59	36	1.4	240	2.1
24	6	2024	12 June 2024	28.1	22.4	16.5	100	69	48	0.0	240	1.9
24	6	2024	13 June 2024	27.0	19.8	14.4	100	85	50	18.4	600	2
24	6	2024	14 June 2024	24.1	18.6	13.9	100	69	38	0.0	360	1.5
24	6	2024	15 June 2024	26.9	20.7	12.0	94	59	36	0.0	60	1.1
25	6	2024	16 June 2024	29.8	23.2	14.0	75	51	35	0.0	0	1.5
25	6	2024	17 June 2024	29.7	22.9	16.1	92	57	28	0.0	60	1.3
25	6	2024	18 June 2024	31.8	23.6	15.1	97	62	33	0.0	60	1.5
25	6	2024	19 June 2024	32.8	24.5	15.8	100	69	33	0.0	300	1.5
25	6	2024	20 June 2024	33.4	25.4	18.0	94	69	46	0.0	60	1.1
25	6	2024	21 June 2024	31.4	26.5	20.9	97	75	57	0.0	0	1.1
25	6	2024	22 June 2024	29.9	25.9	21.2	90	59	34	0.0	0	1.8
26	6	2024	23 June 2024	31.0	24.2	17.1	100	89	68	0.0	840	1.9
26	6	2024	24 June 2024	21.1	19.6	18.0	100	93	79	1.0	720	1.4
26	6	2024	25 June 2024	23.6	19.9	17.6	100	89	68	17.0	660	1.8
26	6	2024	26 June 2024	24.4	20.1	17.1	100	86	58	3.0	540	1.9
26	6	2024	27 June 2024	27.7	21.2	16.7	97	66	39	0.0	60	1.4
26	6	2024	28 June 2024	32.1	25.1	16.9	94	67	42	0.0	0	1.5
26	6	2024	29 June 2024	33.7	26.9	19.7	99	74	47	0.0	180	1.5
27	6	2024	30 June 2024	32.8	26.6	20.0	99	60	32	0.0	120	1.8
27	7	2024	1 July 2024	32.3	26.7	20.9	100	73	44	0.0	660	1.6
27	7	2024	2 July 2024	31.0	24.0	17.2	100	72	37	14.4	540	1.5
27	7	2024	3 July 2024	28.7	22.7	16.5	100	88	61	0.2	660	2
27	7	2024	4 July 2024	23.2	19.8	17.6	100	75	41	11.6	360	2.2
27	7	2024	5 July 2024	27.8	21.6	16.0	100	71	40	0.0	420	1.3
27	7	2024	6 July 2024	29.3	23.4	15.9	100	64	33	0.0	360	1.9
28	7	2024	7 July 2024	31.7	25.4	17.2	96	65	41	0.0	180	1.6
28	7	2024	8 July 2024	30.3	25.2	19.4	98	73	44	0.0	120	1.5
28	7	2024	9 July 2024	31.6	26.2	19.9	99	69	42	0.0	120	1.5
28	7	2024	10 July 2024	33.5	27.3	20.8	95	63	33	0.0	0	1.3
28	7	2024	11 July 2024	35.6	28.6	20.3	91	56	30	0.0	0	1.5
28	7	2024	12 July 2024	36.7	29.3	21.4	89	59	29	0.0	0	1.3
28	7	2024	13 July 2024	34.3	28.1	21.1	79	54	31	0.0	0	1.5
29	7	2024	14 July 2024	33.9	27.7	21.7	86	54	32	0.0	0	1.5
29	7	2024	15 July 2024	33.5	26.4	18.3	90	58	28	0.0	0	1.3
29	7	2024	16 July 2024	34.5	27.5	19.2	88	48	29	0.0	0	1.5
29	7	2024	17 July 2024	35.7	28.1	19.8	81	52	24	0.0	0	1.4
29	7	2024	18 July 2024	35.4	28.2	19.8	83	58	34	0.0	0	1.5
29	7	2024	19 July 2024	34.9	28.1	19.3	94	65	37	0.0	0	1.3
29	7	2024	20 July 2024	35.4	28.6	21.8	100	75	46	0.0	300	1.5
30	7	2024	21 July 2024	31.5	25.5	19.3	100	72	39	26.0	300	1.8
30	7	2024	22 July 2024	32.9	27.1	19.6	100	77	53	0.0	360	1.4
30	7	2024	23 July 2024	32.3	25.4	21.0	100	71	40	0.6	360	1.6
30	7	2024	24 July 2024	32.7	26.5	20.2	97	67	43	0.0	0	1.2
30	7	2024	25 July 2024	32.5	26.8	20.6	98	69	38	0.0	60	1.6
30	7	2024	26 July 2024	31.9	25.8	20.5	94	61	32	0.0	0	2
30	7	2024	27 July 2024	32.7	26.4	17.9	92	66	36	0.0	0	1.8
31	7	2024	28 July 2024	34.8	27.4	19.5	91	60	34	0.0	0	1.3
31	7	2024	29 July 2024	35.9	28.9	20.9	99	71	47	0.0	60	1.8
31	7	2024	30 July 2024	32.8	27.4	22.4	98	61	35	0.0	120	1.6
31	7	2024	31 July 2024	33.1	27.0	20.3	87	58	34	0.0	0	1.6
31	8	2024	1 August 2024	35.9	27.8	19.0	88	58	25	0.0	0	1.2
31	8	2024	2 August 2024	36.2	28.3	21.5	100	79	44	1.0	540	1.6
31	8	2024	3 August 2024	34.1	26.8	20.6	100	84	58	0.0	480	1.4
32	8	2024	4 August 2024	30.1	25.3	20.7	100	70	36	0.0	420	1.7
32	8	2024	5 August 2024	32.9	26.1	19.3	97	66	33	0.0	240	1.5
32	8	2024	6 August 2024	33.5	27.1	19.2	94	69	41	0.0	240	1.8
32	8	2024	7 August 2024	33.4	27.1	20.6	100	73	39	0.0	300	1.6
32	8	2024	8 August 2024	34.8	25.8	19.7	100	72	39	22.8	420	1.7
32	8	2024	9 August 2024	32.0	25.4	19.2	100	69	35	0.0	360	1.3
32	8	2024	10 August 2024	33.9	27.2	20.5	93	64	39	0.0	60	1.1
33	8	2024	11 August 2024	34.9	28.1	21.2	93	60	32	0.0	60	1.2
33	8	2024	12 August 2024	35.9	28.6	21.3	87	57	29	0.0	0	1.1
33	8	2024	13 August 2024	37.2	29.5	21.4	83	57	30	0.0	0	1.8
33	8	2024	14 August 2024	37.7	29.6	22.6	80	61	31	0.0	0	1.6
33	8	2024	15 August 2024	36.3	26.9	20.5	92	67	48	33.4	60	0.9
33	8	2024	16 August 2024	30.5	25.3	20.3	86	64	42	0.0	0	1
33	8	2024	17 August 2024	33.0	26.0	21.7	100	68	32	0.0	300	1.1
34	8	2024	18 August 2024	33.3	25.8	21.4	100	81	43	3.6	540	1.1
34	8	2024	19 August 2024	30.0	23.8	19.9	100	97	86	13.2	720	0.8
34	8	2024	20 August 2024	23.5	21.5	19.6	100	93	64	17.8	540	1.2
34	8	2024	21 August 2024	28.4	23.8	20.7	100	76	44	11.2	120	1
34	8	2024	22 August 2024	32.5	26.1	19.8	100	82	55	0.0	420	1.8
34	8	2024	23 August 2024	31.0	25.7	20.2	100	79	48	0.0	420	1.7
34	8	2024	24 August 2024	33.8	26.6	20.8	99	70	37	0.0	60	1.7
35	8	2024	25 August 2024	34.3	27.2	19.9	84	57	26	0.0	0	1.2
35	8	2024	26 August 2024	34.8	27.2	19.6	92	70	46	0.0	0	1.3
35	8	2024	27 August 2024	32.9	26.2	20.7	100	87	57	0.0	540	0.5

S: week. M: month. TMx: maximum daily temperature, °C. TAv: average daily temperature, °C. TMn: minimum daily temperature, °C. RH Mx: maximum daily relative humidity, %. RH Av: average daily relative humidity, %. RH Mn: minimum daily relative humidity, %. Rainfall: daily rainfall, mm/m^2.^ Leaf Wetness: minute daily leaf wetness. Wind: average daily wind, m/s.

**Table 6:** Summary table of transplanting and time intervals between applications.

Experiment	Transplanting	1st Application (dpt)	2nd Application Interval (d)	3rd Application Interval (d)
1	23 May 2023	15	30	30
2a	20 May 2024	15	30	30
2b	20 May 2024	15	20	20
3	9 May 2024	30	20	20

dpt, days post-transplant. d, days.

## Data Availability

The original contributions presented in this study are included in the article/supplementary material. Further inquiries can be directed to the corresponding author.

## Supplementary Materials

The following supporting information can be downloaded at https://www.mdpi.com/article/10.3390/plants14152411/s1: Figure S1: Figures comparing stomatal patterns under varying salinity stress conditions; Figure S2: Total (A) and marketable (B) production of plants treated with 100 fM of PS1-70 and G1 in San Giovanni in Persiceto, Bologna (Italy), 2023 (Experiment 1); Figure S3: Total (A) and marketable (B) production of plants treated with 100 fM solutions of PS1-70 and G1 in Lagosanto, Ferrara, 2024, in Experiment 2b, in which plants were treated every 20 days; Figure S4: Total (A) and marketable (B) production of plants treated with 100 fM solutions of PS1-70 and G1 in Vedrana (BO), 2024 (Experiment 3), for plants treated every 20 days; Table S1: Gross marketable production (GMP) of plants treated with 100 fM solutions of PS1-70 and G1 across 2023 (Experiment 1 in San Giovanni in Persiceto, Bologna) and 2024 trials (Experiment 2a and 2b in Lagosanto, Ferrara and Experiment 3 in Vedrana, Bologna); Table S2: °Brix of tomato fruits from plants treated with 100 fM solutions of PS1-70 and G1 measures in 2023 (Experiment 1 in San Giovanni in Persiceto, Bologna, Italy) and during 2024 trials (Experiment 2a, 2b, in Lagosanto, Ferrara, and Experiment 3 in Vedrana, Bologna, Italy); Table S3: List of defense genes and specific primers used for expression analysis; Table S4: Detailed values of marketable production for each replicate per treatment of Experiment 1 (San Giovanni in Persiceto, 2023). Letters indicate replicates; Table S5: Detailed values of total production for each replicate per treatment of Experiment 1 (San Giovanni in Persiceto, 2023). Letters indicate replicates; Table S6: Detailed values of gross marketable production for each replicate per treatment of Experiment 1 (San Giovanni in Persiceto, BO. 2023). Letters indicate replicates; Table S7: Detailed values of °Brix for each replicate per treatment of experiment 1 (San Giovanni in Persiceto, 2023). Letters indicate replicates; Table S8: Detailed values of marketable production for each replicate per treatment of Experiment 2a (Lagosanto, 2024). Letters indicate replicates; Table S9: Detailed values of total production for each replicate per treatment of Experiment 2a (Lagosanto, 2024). Letters indicate replicates; Table S10: Detailed values of total gross marketable production for each replicate per treatment of Experiment 2a (Lagosanto, 2024). Letters indicate replicates; Table S11: Detailed values of total °Brix for each replicate per treatment of Experiment 2a (Lagosanto, 2024). Letters indicate replicates; Table S12: Detailed values of marketable production for each replicate per treatment of Experiment 2b (Lagosanto, 2024). Letters indicate replicates; Table S13: Detailed values of total production for each replicate per treatment of Experiment 2b (Lagosanto, 2024). Letters indicate replicates; Table S14: Detailed values of gross marketable production for each replicate per treatment of Experiment 2b (Lagosanto, 2024). Letters indicate replicates; Table S15: Detailed values of total °Brix for each replicate per treatment of Experiment 2b (Lagosanto, 2024). Letters indicate replicates; Table S16: Detailed values of marketable production for each replicate per treatment of Experiment 3 (Vedrana (Budrio), 2024). Letters indicate replicates; Table S17: Detailed values of total production for each replicate per treatment of Experiment 3 (Vedrana (Budrio), 2024). Letters indicate replicates; Table S18: Detailed values of gross marketable production for each replicate per treatment of Experiment 3 (Vedrana (Budrio), 2024). Letters indicate replicates; Table S19: Detailed values of total °Brix for each replicate per treatment of Experiment 3 (Vedrana, Budrio, 2024). Letters indicate replicates.

## Abbreviations

The following abbreviations are used in this manuscript:

APX	Ascorbate peroxidase
CAT2	Catalase
CDPKs	Calcium-dependent protein kinase
CMs	Calmodulins
CV	Coefficient of variation
EFs	Experimental fragments
FW	Fresh weight
GEP	Good experimental practice
GMP	Gross marketable production
HSP90	Heat shock protein
JA	Jasmonic acid
LRR-RK	Leucine-rich repeat receptor kinase
ProSys	Prosystemin
RMs	Repeat motifs
ROS	Reactive oxygen species
SA	Stomatal area
SD	Stomatal density
SFW	Shoot fresh weight
Sys	Systemin
VOCs	Volatile organic compounds
