# Supplementary material for: Salt Stress Mitigation and Field-Relevant Biostimulant Activity of Prosystemin Protein Fragments: Novel Tools for Cutting-Edge Solutions in Agriculture
Source: Plants (Basel). 2025 Aug 4;14(15):2411. doi: 10.3390/plants14152411 (PMC12349507; doi:10.3390/plants14152411)
Supplement: Supplementary file 1 [file plants-14-02411-s001.zip › plants-3740822-supplementary.pdf]

SUPPLEMENTARY FILE

Salt stress mitigation and field-relevant biostimulant activity of Prosystemin protein fragments: novel tools for cutting-edge solutions in agriculture

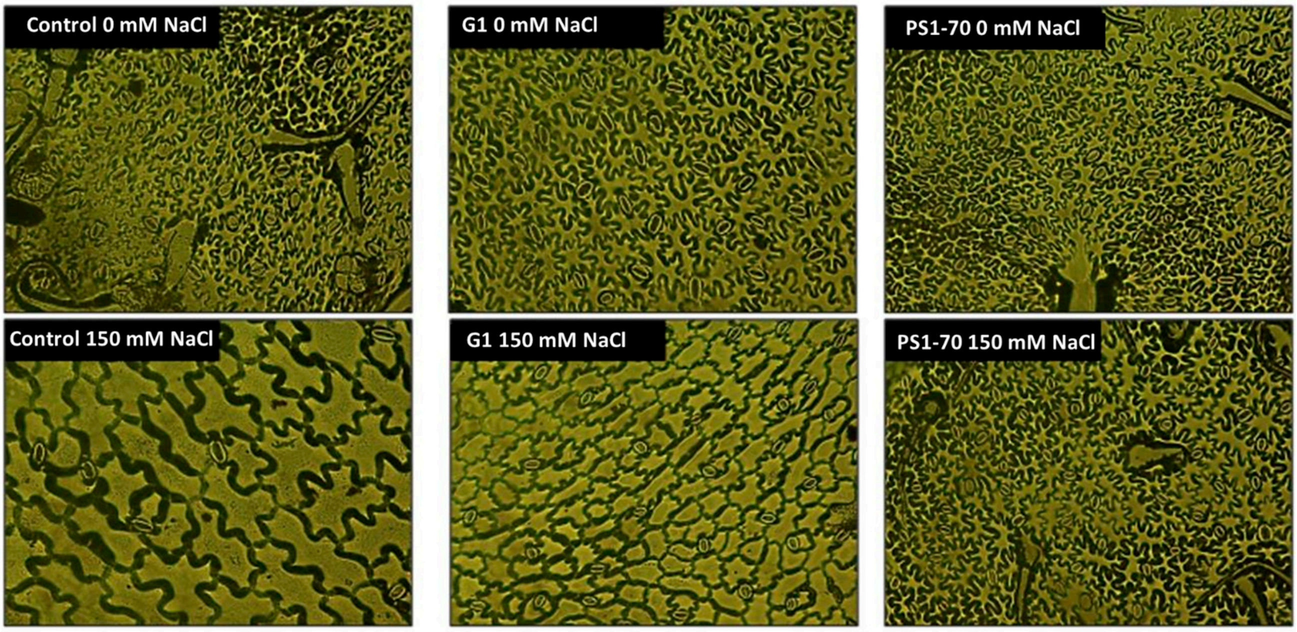

Supplementary Figure S1. Figures comparing stomatal patterns under varying salinity stress conditions. Microscopic images show the epidermal structure of leaves from different treatments: Control (0 mM NaCl), G1 (0 mM NaCl), and PS1-70 (0 mM NaCl) for non-stress conditions (top row) and Control (150 mM NaCl), G1 (150 mM NaCl), and PS1-70 (150 mM NaCl) for stress conditions (bottom row). The images reveal significant changes in stomatal patterns and density when exposed to high salinity (150 mM NaCl) across all treatments.

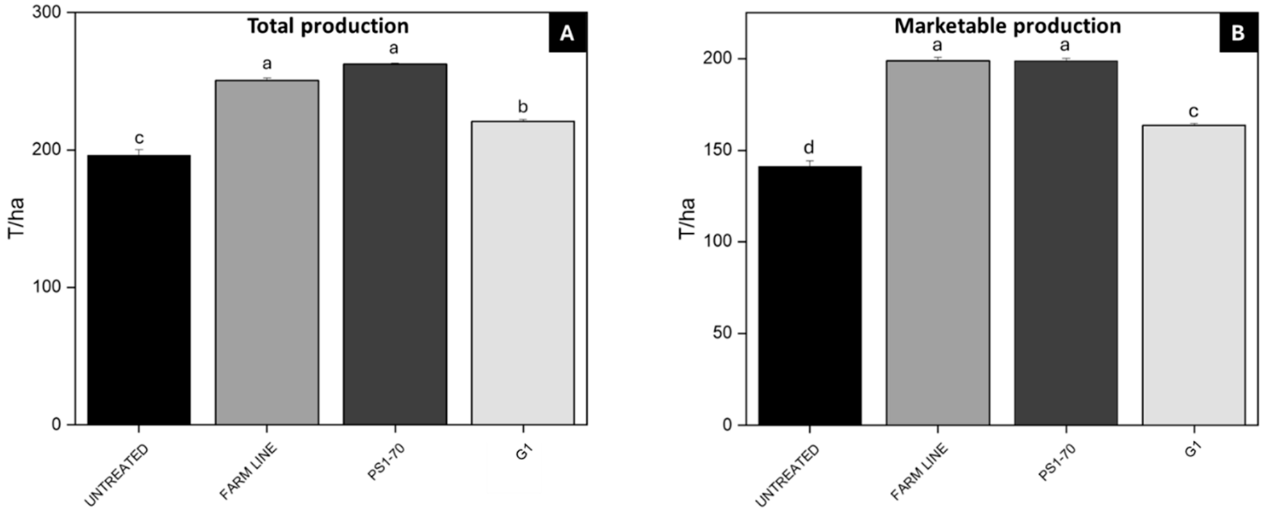

Supplementary Figure S2: Total (A) and marketable (B) production of plants treated with 100 fM of PS1-70 and G1 conducted in San Giovanni in Persiceto, Bologna (Italy), 2023 (Experiment 1). Error bars indicate standard error). Means were compared using the Student-Newman-Keuls (SNK) test ($P \leq 0.05$). Letters indicate statistically significant differences between the experimental groups.

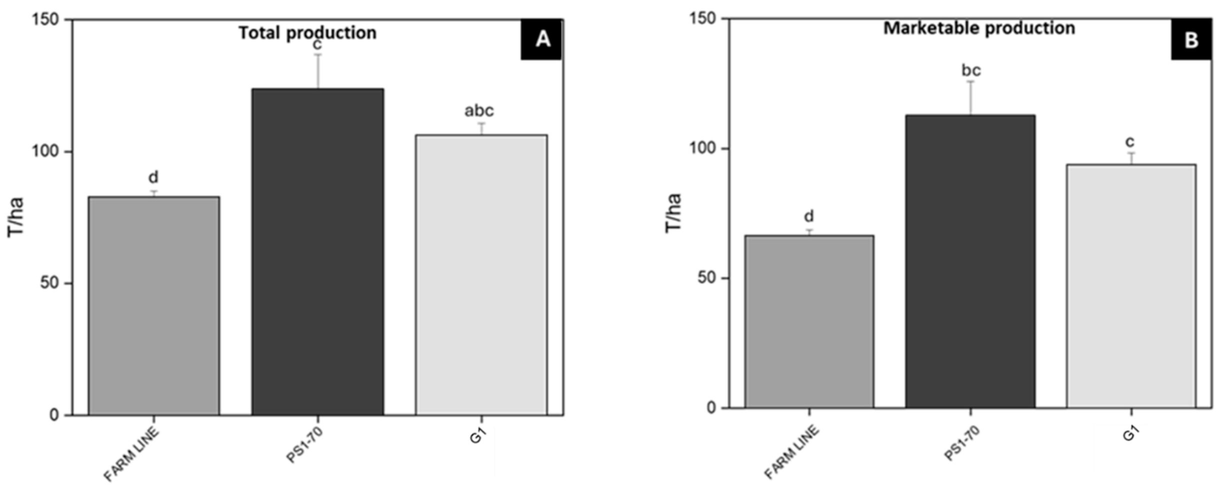

Supplementary Figure S3: Total (A) and marketable (B) production of plants treated with 100 fM solutions of PS1-70 and G1 in Lagosanto, Ferrara, 2024 of Experiment 2b treating plants every 20 days. Error bars indicate standard error (n=4). Means were compared using the Student-Newman-Keuls (SNK) test ($P \leq 0.05$). Letters indicate statistically significant differences between the experimental groups.

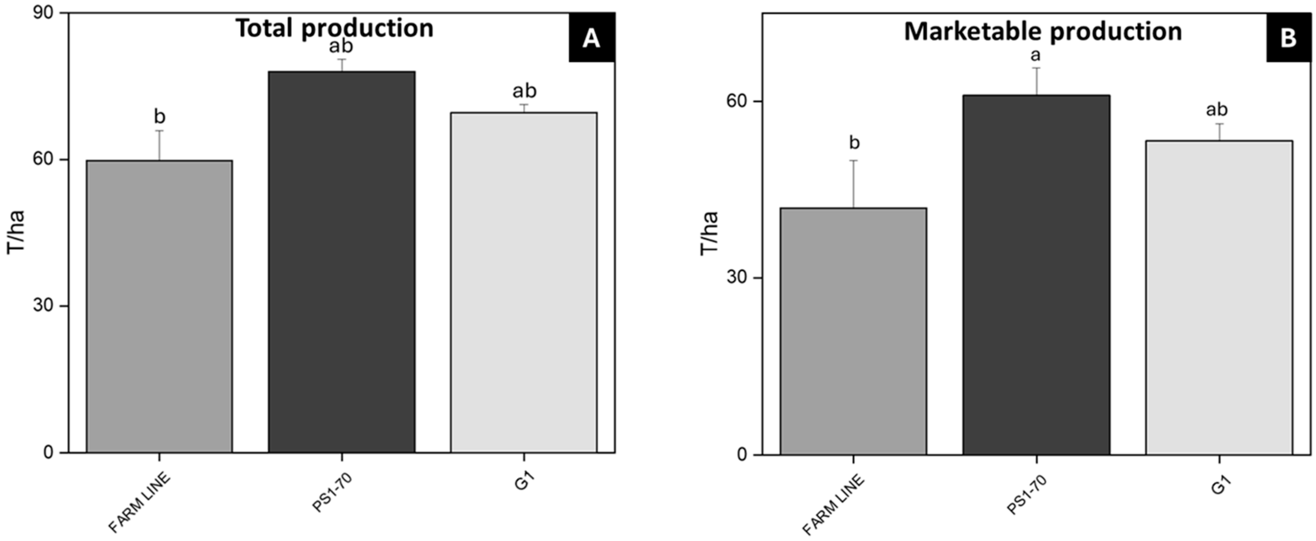

Supplementary Figure S4: Total (A) and marketable (B) production of plants treated with 100 fM solutions of PS1-70 and G1 in Vedrana (BO), 2024 (Experiment 3) treating plant every 20 days. Error bars indicate standard error (n=4). Means were compared using the Student-Newman-Keuls (SNK) test ($P \leq 0.05$). Letters indicate statistically significant differences between the experimental groups.

Supplementary Table S1: Gross marketable production (GMP) of plants treated with 100 fM solutions of PS1-70 and G1 across 2023 (Experiment 1 in San Giovanni in Persiceto, Bologna), 2024 trials (Experiment 2b in Lagosanto, Ferrara and Experiment 3 in Vedrana, Bologna). The values are mean ± standard error (n=4). Means were compared using the Student-Newman-Keuls (SNK) test ($P \leq 0.05$). Letters indicate statistically significant differences between the experimental groups.

Trials	Treatments	GMP (€/ha)
**2023 trial- Experiment 1**	Farm line	35058.33a
	PS1-70	36750.00a
	G1	30916.67b
**2024 trial- Experiment 2b**	Farm line	9310.0d
	PS1-70	15796.7abc
	G1	13136.7c
**2024 trial - Experiment 3**	Farm line	5862.5b
	PS1-70	8545.8a
	G1	7466.7a

Supplementary Table S2: °Brix of tomato fruits from plants treated with 100 fM solutions of PS1-70 and G1 measures in 2023 (Experiment 1 in San Giovanni in Persiceto, Bologna, Italy) and during 2024 trials (Experiment 2a, 2b, in Lagosanto, Ferrara, and Experiment 3 in Vedrana, Bologna, Italy).

**Trials**	**Treatments**	**°Brix**
**2023 trial- Experiment 1**	Untreated	4.70
	Farm line	5.00
	PS1-70	4.90
	G1	4.90
**2024 trial- Experiment 2a**	Farm line	5.1
	PS1-70	5.4
	G1	4.9
**2024 trial- Experiment 2b**	Farm line	5.1
	PS1-70	5.1
	G1	4.8
**2024 trial- Experiment 3**	Farm line	4.8
	PS1-70	4.9
	G1	4.9

Supplementary Table S3: List of defence genes and specific primers used for expression analysis.

**Gene**	**Accession**	**5′-3′**	**T_m[1]**
EF1-α	X53043	Fw: AAGCTGCTGAGATGAACAAG	58
		Rv: TCAAACCAGTAGGGCCAAA	54
CAT2	AF112368	Fw: GCACAGGGATGAGGAGATCG	59
		Rv: TCTGTCGGGTGTGAATGAGC	55
APX2	DQ099421	Fw: TGGGAGGGTGGTGACATATTTT	57
		Rv: TTGAAGTGCATAACTTCCCATCTTT	53
HSP90	AF123259	Fw: GGGCCCATGTCGGACGTAGAGACGTTTG	58
		Rv: CCCGGGTTAATCAACCTCCTCCATCTTGCT	55
AOS	Q9LLB0	Fw: GATCGGTTCGTCGGAGAAGAA	58.9
		Rv: GCGCACTGTTTATTCCCCACT	60
Pin I	P05118	Fw: GAAACTCTCATGGCACGAAAAG	57.2
		Rv: CACCAATAAGTTCTGGCCACAT	57.9
Pin II	A0A3Q7FEG3	Fw: CCAAAAAGGCCAAATGCTTG	55.7
		Rv: TGTGCAACACGTGGTACATCC	60.2

Tm[1]: temperature of melting calculated according to Wallace's rule: 4ºC for G and C, 2ºC for A and T (Wallace et al., 1979).

Supplementary Table S4: Detailed values of marketable production for each replicate per treatment of Experiment 1 (San Giovanni in Persiceto, 2023). Letters indicate replicates.

	Marketable plot production T/ha				
**Thesis**	**A**	**B**	**C**	**D**	**Average**
**UNTREATED control**	148,33	143,33	140,00	133,33	**141,25**
**FARM LINE**	203,33	200,00	198,33	193,33	**198,75**
**PS1-70**	198,33	200,00	201,67	195,00	**198,75**
**G1**	166,67	163,33	161,67	163,33	**163,75**

Supplementary Table S5: Detailed values of total production for each replicate per treatment of Experiment 1 (San Giovanni in Persiceto, 2023). Letters indicate replicates.

	Total plot production T/ha				
**Thesis**	**A**	**B**	**C**	**D**	**Average**
**UNTREATED control**	203,33	200,00	196,67	183,33	**195,83**
**FARM LINE**	253,33	253,33	250,00	245,00	**250,42**
**PS1-70**	263,33	263,33	263,33	260,00	**262,50**
**G1**	223,33	223,33	218,33	218,33	**220,83**

Supplementary Table S6: Detailed values of Gross Marketable Production for each replicate per treatment of Experiment 1 (San Giovanni in Persiceto, BO. 2023). Letters indicate replicates.

	Gross Marketable Production €/ha				
**Thesis**	**A**	**B**	**C**	**D**	**Average**
**UNTREATED control**	28466,67	28000,00	27533,33	25666,67	**27416,67**
**FARM LINE**	35466,67	35466,67	35000,00	34300,00	**35058,33**
**PS1-70**	36866,67	36866,67	36866,67	36400,00	**36750,00**
**G1**	31266,67	31266,67	30566,67	30566,67	**30916,67**

Supplementary Table S7: Detailed values of ° brix for each replicate per treatment of Experiment 1 (San Giovanni in Persiceto, 2023). Letters indicate replicates.

	Brix value				
**Thesis**	**A**	**B**	**C**	**D**	**Average**
**UNTREATED control**	4,90	4,80	4,60	4,50	**4,70**
**FARM LINE**	5,00	4,90	4,80	5,30	**5,00**
**PS1-70**	4,80	4,70	4,90	5,20	**4,90**
**G1**	4,80	4,60	5,00	5,20	**4,90**

Supplementary Table S8: Detailed values of marketable production for each replicate per treatment of Experiment 2a (Lagosanto,2024). Letters indicate replicates.

	Marketable plot production T/ha				
**Thesis**	**A**	**B**	**C**	**D**	**Average**
**FARM LINE**	63,33	70,00	66,67	66,00	**66,50**
**G1 30 days**	103,33	113,33	120,00	116,00	**113,17**
**PS1-70 30 days**	78,67	108,00	123,33	101,33	**102,83**

Supplementary Table S9: Detailed values of total production for each replicate per treatment of Experiment 2a (Lagosanto,2024). Letters indicate replicates.

	Total plot production T/ha				
**Thesis**	**A**	**B**	**C**	**D**	**Average**
**FARM LINE**	76,67	85,33	86,67	82,67	**82,83**
**G1 30 days**	118,67	126,67	135,33	130,00	**127,67**
**PS1-70 30 days**	93,33	122,00	136,67	114,00	**116,50**

Supplementary Table S10: Detailed values of total Gross Marketable Production for each replicate per treatment of Experiment 2a (Lagosanto,2024). Letters indicate replicates.

	Gross Marketable Production €/ha				
**Thesis**	**A**	**B**	**C**	**D**	**Average**
**FARM LINE**	8866,7	9800,0	9333,3	9240,0	**9310,0**
**G1 30 days**	14466,7	15866,7	16800,0	16240,0	**15843,3**
**PS1-70 30 days**	11013,3	15120,0	17266,7	14186,7	**14396,7**

Supplementary Table S11: Detailed values of total °brix for each replicate per treatment of Experiment 2a (Lagosanto,2024). Letters indicate replicates.

	Brix value				
**Thesis**	**A**	**B**	**C**	**D**	**Average**
**FARM LINE**	5,10	5,00	4,90	5,40	**5,1**
**G1 30 days**	4,80	5,00	5,10	4,70	**4,9**
**PS1-70 30 days**	5,50	5,20	5,30	5,60	**5,4**

Supplementary Table S12: Detailed values of marketable production for each replicate per treatment of Experiment 2b (Lagosanto,2024). Letters indicate replicates.

	Marketable plot production T/ha				
**Thesis**	**A**	**B**	**C**	**D**	**Average**
**FARM LINE**	63,33	70,00	66,67	66,00	**66,50**
**G1 20 days**	86,67	101,33	93,33	94,00	**93,83**
**PS1-70 20 days**	76,67	124,67	133,33	116,67	**112,83**

Supplementary Table S13: Detailed values of total production for each replicate per treatment of Experiment 2b (Lagosanto,2024). Letters indicate replicates.

	Total plot production T/ha				
**Thesis**	**A**	**B**	**C**	**D**	**Average**
**FARM LINE**	76,67	85,33	86,67	82,67	**82,83**
**G1 20 days**	93,33	108,67	113,33	110,00	**106,33**
**PS1-70 20 days**	86,67	132,67	146,67	129,33	**123,83**

Supplementary Table S14: Detailed values of Gross Marketable Production for each replicate per treatment of Experiment 2b (Lagosanto,2024). Letters indicate replicates.

	Gross Marketable Production €/ha				
**Thesis**	**A**	**B**	**C**	**D**	**Average**
**FARM LINE**	8866,7	9800,0	9333,3	9240,0	**9310,0**
**G1 20 days**	12133,3	14186,7	13066,7	13160,0	**13136,7**
**PS1-70 20 days**	10733,3	17453,3	18666,7	16333,3	**15796,7**

Supplementary Table S15: Detailed values of total °brix for each replicate per treatment of Experiment 2b (Lagosanto,2024). Letters indicate replicates.

	Brix value				
**Thesis**	**A**	**B**	**C**	**D**	**Average**
**FARM LINE**	5,10	5,00	4,90	5,40	**5,1**
**G1 20 days**	4,90	4,80	4,70	4,80	**4,8**
**PS1-70 20 days**	5,00	5,20	4,90	5,30	**5,1**

Supplementary Table S16: Detailed values of marketable production for each replicate per treatment of Experiment 3 (Vedrana, Budrio, 2024). Letters indicate replicates.

	Marketable plot production t/ha				
**Thesis**	**A**	**B**	**C**	**D**	**Average**
**FARM LINE**	24,17	50,00	43,33	50,00	**41,88**
**G1**	50,83	48,33	60,00	54,17	**53,33**
**PS1-70**	63,33	60,00	56,67	64,17	**61,04**

Supplementary Table S17: Detailed values of total production for each replicate per treatment of Experiment 3 (Vedrana, Budrio, 2024). Letters indicate replicates.

	Total plot production t/ha				
**Thesis**	**A**	**B**	**C**	**D**	**Average**
**FARM LINE**	35,83	66,67	65,00	71,67	**59,79**
**G1**	59,17	66,67	81,67	70,83	**69,58**
**PS1-70**	76,33	84,00	70,83	80,83	**78,00**

Supplementary Table S18: Detailed values of Gross Marketable Production for each replicate per treatment of Experiment 3 (Vedrana, Budrio, 2024). Letters indicate replicates.

	Gross Marketable Production €/ha				
**Thesis**	**A**	**B**	**C**	**D**	**Average**
**1FARM LINE**	3383,33	7000,00	6066,67	7000,00	**5862,50**
**G1**	7116,67	6766,67	8400,00	7583,33	**7466,67**
**PS1-70**	8866,67	8400,00	7933,33	8983,33	**8545,83**

Supplementary Table S19: Detailed values of total °brix for each replicate per treatment of Experiment 3 (Vedrana, Budrio, 2024). Letters indicate replicates.

	Brix value				
**Thesis**	**A**	**B**	**C**	**D**	**Average**
**FARM LINE**	4,80	4,90	5,00	4,50	**4,8**
**G1**	4,90	4,80	5,10	4,80	**4,9**
**PS1-70**	5,00	4,90	4,70	5,00	**4,9**
